# The Impact of Microbiota-Gut-Brain Axis on Diabetic Cognition Impairment

**DOI:** 10.3389/fnagi.2017.00106

**Published:** 2017-04-27

**Authors:** Youhua Xu, Hua Zhou, Quan Zhu

**Affiliations:** ^1^Faculty of Chinese Medicine, Macau University of Science and TechnologyTaipa, Macau; ^2^State Key Laboratory of Quality Research in Chinese Medicine (Macau University of Science and Technology)Taipa, Macau; ^3^Laboratory for Bioassay and Molecular Pharmacology of Chinese Medicines, Macau Institute for Applied Research in Medicine and HealthTaipa, Macau; ^4^Guangdong Consun Pharmaceutical Group, Institute of Consun Co. for Chinese Medicine in Kidney DiseasesGuangzhou, China

**Keywords:** advanced glycation end products, diabetic encephalopathy, hypothalamic-pituitary-adrenal axis, inflammation, microbiota, gut

## Abstract

Progressive cognitive dysfunction is a central characteristic of diabetic encephalopathy (DE). With an aging population, the incidence of DE is rising and it has become a major threat that seriously affects public health. Studies within this decade have indicated the important role of risk factors such as oxidative stress and inflammation on the development of cognitive impairment. With the recognition of the two-way communication between gut and brain, recent investigation suggests that “microbiota-gut-brain axis” also plays a pivotal role in modulating both cognition function and endocrine stability. This review aims to systemically elucidate the underlying impact of diabetes on cognitive impairment.

## Introduction

Diabetic neuropathy refers to a series of neurological dysfunction caused by diabetes. With the duration of the disease, the nerve damage may occur throughout the body of diabetic patient. In Miles and Root (1922) noticed that diabetes can cause central nervous system lesions which will finally result in cognitive dysfunction. In Reske-Nielsen et al. (1966) proposed the concept “diabetic encephalopathy (DE)” when they studied the brain tissues from 16 young diabetic patients who died of vascular complications. Due to the lack of uniform diagnostic criteria, epidemiological survey concerning DE is very difficult, but it is suggested that the incidence of cognitive impairment in the diabetic population may reach as high as 40%. A clinical cohort study suggested that brain atrophy is significantly and positively correlated with type 2 diabetes (Wisse et al., 2014). Progressive cognitive impairment is a central characteristic of DE. A recent meta-analysis that included 1,148,041 cases found that diabetes can increase the risk of cognitive impairment by about 2 times (Gudala et al., 2013). To date, progressive cognitive dysfunction has been recognized as a typical signs and symptoms in diabetic population (van den Berg et al., 2010).

With an aging population, the incidence of diabetes is rising; more importantly, more and more DE cases are found in younger population. The damage attributed to diabetes on cognitive function has become a major threat that seriously affects the quality of life. Therefore, speeding up the study of the pathogenesis of diabetic cognitive impairment (DCI) and establishing an effective prevention strategy is urgent. There is a study found that cognitive dysfunction may occur in the early stage of diabetes and will progress with the disease, and this progression is much faster in type 2 diabetes than that in type 1 patients (Brands et al., 2007). In this sense, early intervention is very important.

As a metabolic disease, the development of diabetes is dually controlled and regulated by neuroendocrine factors and digestive system. With the deepening of the study, it is suggested that gut-brain crosstalk may play a key role in this process. Gut-brain crosstalk is a very complex network system; it maintains the stability of gastrointestinal tract on the one hand and affects the emotion and cognition function on the other hand, and this network is known as “gut-brain axis (GBA)” (Rhee et al., 2009). Recent studies indicated that there is a two-way communication between gut and brain. Bercik et al. (2011) demonstrated that gut microbes can affect the level of rat brain-derived neurotrophic factor; Dinan and Cryan (2012) found stress may in turn activate the hypothalamus by affecting the intestinal microbial activity—pituitary—adrenal axis which will lead to depression. Another research conducted in animals also observed that germ-free can impair memory function (Gareau et al., 2011). At present, it is believed that gut **m**icrobes-**g**ut-**b**rain **a**xis (MGBA) may be an ideal target for understanding and treating DCI (Foster and McVey Neufeld, 2013).

## The pathogenesis of diabetic encephalopathy

Diabetic cognitive impairment (DCI) refers to cognitive impairment and brain physiological and structural changes caused by diabetes. Both type 1 and type 2 diabetes can induce and promote DCI development (Biessels et al., 2002a). There is a study observed that the characteristics of neurobiology and neuroradiological imaging of DCI is very similar to that of brain aging (Biessels et al., 2002b), suggesting DCI shares similar mechanisms with the brain aging process.

The pathogenesis of DCI has not yet been entirely clarified, but it is found that cerebral ischemia, oxidative stress, and non-enzymatic protein glycosylation, low grade inflammation and calcium homeostasis changes, etc., may play a role in the development of DCI.

### Cerebral vascular dysfunction

Blood-brain barrier (BBB) is located on the nerve endometrial capillaries, including peripheral nerve microvascular endothelial cells (PnMECs), pericytes of endoneurial microvascular origin, and basement membrane (Poduslo et al., 1994; Abbott et al., 2006). The physiological function of BBB is realized by physical barrier and the ionic charge on the cerebral vascular endothelial cells, and any changes will lead to BBB dysfunction. At present, the hypertrophy of basement membrane and the lysis of BBB have been recognized as characteristic changes in diabetic neuropathy (Giannini and Dyck, 1995; Shimizu et al., 2011).

In fact, BBB damage has now been considered as a key factor for DCI (Biessels et al., 2008). It has been demonstrated that pathological changes associated with type 2 diabetes can damage BBB integrity, increase the permeability of BBB and lead to the easy permeation of limited substances into brain parenchyma (Dai et al., 2002; Hawkins et al., 2007; Kamada et al., 2007). A recent research demonstrated that BBB breakdown promotes the macrophage infiltration and cognition impairment in mice (Stranahan et al., 2016), suggesting BBB dysfunction is closely related with diabetic cerebral inflammation.

#### Physical barrier of BBB

The physical barrier of BBB is composed of two components, i.e.,: vascular endothelial cells and basement membrane. Studies have demonstrated that diabetes and continuous high blood glucose will directly and finally induce dramatic damage of endothelial cells in both cerebral and peripheral vascular system (Xu et al., 2010; Li et al., 2016), and chronic untreated diabetes would also impair BBB by reducing tight junction proteins (e.g., ZO-1 and claudin-5) expressions and thus cause a series of cerebral dysfunction (Yoo et al., 2016). This is confirmed from a research that diabetes will significantly increase cerebral vascular permeability (Fouyas et al., 2003).

Enough blood supply plays a pivotal role in maintaining the normal function of brain. Diabetes can significantly induce cerebral vascular endothelial dysfunction and increase platelet aggregation, reduce cerebral blood flow, and cerebral vascular surface area, and finally lead to vascular endothelial proliferation and plasma viscosity increment (Dalal and Parab, 2002; Fouyas et al., 2003). Recently, Yu et al. (2016) found in diabetic patients that the disruption of BBB is significantly associated with acute stroke. Therefore, protecting the integrity of cerebral vascular endothelial cells should have important effects on reducing diabetic damage to the brain.

The basement membrane of BBB is composed of extracellular matrix adhesion proteins (e.g., type IV collagen) and fibronectin, and is strictly regulated by matrix metalloproteinases (MMPs); studies found MMP-2 and MMP-9 can degrade type IV collagen and fibronectin (Tilling et al., 2002; Chang, 2016). In a most recently published clinical study, Garro et al. (2017) found that the blood MMP-2 is lower while MMP-9 is higher in children with diabetic ketoacidosis (DKA) compared with levels in children without DKA, strongly suggesting the important role of BBB in the development of DE.

#### Charge barrier changes in BBB under diabetic condition

Concerning the charge barrier changes under diabetes, amounts of studies have demonstrated that there is negative relation between blood glucose and anionic charge levels on the cell membrane. This relation is most obvious in patients with diabetic nephropathy (Márquez et al., 2015). As well known, heparan sulfate is a negatively charged polysaccharide that is abundantly expressed in all layers of the glomerular filtration barrier (GFB), therefore, it is believed to play a central role in the development of diabetic proteinuria (Garsen et al., 2014). In fact, more and more studies have also reported this correlation in DCI. Previously, Briani et al. (2002) found that titers of heparin sulfate antibodies are elevated in neurological associated disease and concluded that it might associate with the breakdown of BBB. Recent studies confirmed that heparan sulfate proteoglycan agrin accumulation is related with the maturation of BBB during embryogenesis, and agrin contributes to brain endothelium tight junctions (Steiner et al., 2014) and endfoot membrane integrity of astrocytes (Noell et al., 2009).

### Nitrogen/oxygen stress and non-enzymatic glycosylation

#### Oxidative stress damage of DCI

Glucose metabolism disorder is one of the basic reasons for diabetic damage. Blood glucose is the main energy source of the brain and mitochondria is the most important place for glucose aerobic oxidation in the brain. Under chronic and persistent high glucose condition, mitochondria will produce large amounts of reactive oxygen species (ROS) and leads to the oxidative stress, which will impair mitochondrial function and finally affect brain function (Liu et al., 2006; Cardoso et al., 2013). Reports have confirmed that hippocampus and cerebral cortex show a significant oxidative stress in DCI (Grillo et al., 2003; Mastrocola et al., 2005) and anti-oxidative stress treatment is believed to have a positive effect on ameliorating cognitive impairment (Kuhad and Chopra, 2007).

Chronic and sustained high glucose and ROS stimulation can directly stimulate apoptosis of neuronal cells (Liu et al., 2003). As discussed above, changes in BBB, including the integrity of the BBB damage and increased permeability, may cause diabetic cognitive dysfunction. In fact, ROS can increase the BBB permeability by down-regulating expression of tight junction proteins and remodeling cerebral vascular structure. A research demonstrated in human-source highly immortalized brain endothelial cell line hCMEC/D3 that ROS can activate PI3K-PKB pathway, induce cytoskeletal actin rearrangement and spatial redistribution, suppress tight junction proteins expression, and finally increase cerebral endothelial cell permeability and alter the integrity of BBB (Schreibelt et al., 2007).

On the other hand, oxidative stress can also impair neurogenesis. There are two locations existing immortalized neural stem cells in the brain of mammalian species: namely the subventricular zone (SVZ) and the subgranular zone (SGZ). The nerve cells generated from these two sites can be integrated into the local nervous loop and participate in learning and memory processes. Study from Edgardo and colleagues (Alvarez et al., 2009) confirmed that the learning and memory ability is dramatically decreased in STZ-induced diabetic mice compared with normal mice; the DCX-positive cells, which reflect the amounts of new-born neurons, are significantly reduced in diabetic animal; and the lipofuscin deposition in SVZ and SGZ is dramatically increased, indicating oxidative stress contributed to the development of neurogenesis disorders.

#### Non-enzymatic protein glycosylation

As discussed above, the excessive oxidative stress has been recognized as one of the most important pathogenesis of diabetes. Studies indicated that the process of oxidative stress is strictly associated with protein glycosylation, and these two synergic factors contribute to the worsening of diabetes and diabetic complications, including diabetic cerebral vascular damage and cognitive obstacles (Vlassara and Palace, 2003). It has been well recognized that the severity of diabetic neuropathy is closely related with the history of diabetes and the level of hyperglycemia (Dahl-Jørgensen et al., 1986). Chronic and sustained high glucose environment will increase the generation of advanced glycation end products (AGEs). Studies suggest that AGEs participate in the whole process of the pathophysiology of diabetic neuropathy. In a prospective clinical study that lasted for 27 years, researchers found that the degree of neuropathy is related with HbA1c and AGEs (Sveen et al., 2013).

AGEs are a broad class of non-enzymatic products of reactions between proteins or lipids and aldose sugars (Singh et al., 2001) characterized by fluorescence, brown color, and intra- and inter-molecular cross-linking and are formed by the process of nonenzymatic glycation, in which reducing sugars such as glucose react non-enzymatically with amino groups of proteins and other macromolecules. In addition to glucose, other reactive dicarbonyls, such as methylglyoxal (Mgx), glyoxal (Gx), and deoxyglucosones, are also known to generate AGEs (Brownlee et al., 1984; Wautier and Guillausseau, 1998). So far, only a few AGE structures have been identified *in vivo*, such as Nε-(carboxymethyl)lysine (CML), pentosidine, imidazolones, and oxalic acid monolysinylamide (OMA) et al. Although AGEs can be continuously produced and accumulated under high glucose circumstance in the body, western diet which is accompanied with high AGEs in the food will dramatically accelerate this process, as about 10% of oral consumed AGEs can be absorbed into the circulation system (Uribarri et al., 2011; Vlassara and Striker, 2011; Illien-Jünger et al., 2015). When studying the relation between Maillard reaction products and Alzheimer's disease, Smith et al. (1994) observed that the level of AGEs at neurofibrillary tangles and senile plaques is significantly increased in the patients. More importantly, AGEs are found to be co-localized with astrocytes and microglia in these patients (Takeda et al., 1998), strongly suggesting the important role of AGEs in DCI development. The composition of AGEs is very complex. There is a study demonstrated that CML, one of the major components of AGEs, is accumulated in the nervous system of diabetic patients (Sugimoto et al., 1997).

Recently, the *in vivo* effect of D-ribose (Rib) on glycosylation has attracted more and more interests (Wei et al., 2012). Rib exists in all kinds of cells and is a key component of many important biological molecules (Keller et al., 1988). There is a clinical study involving type 2 diabetes patients reported that the urine level of Rib in these patients is abnormally high (Tao et al., 2013), and this elevation participates in cognitive dysfunction in these patients (Han et al., 2014). This finding is further demonstrated by animal experiments that intraperitoneal injection of Rib to mice can significantly increase the plasma glycated proteins and AGEs content, while with less impact on blood sugar (Wei et al., 2012); moreover, this treatment significantly increases brain levels of AGEs, and contributes to learning and memory decline (Han et al., 2011).

AGEs can alter protein features and affect cell function via multiple pathways (Duran-Jimenez et al., 2009). Although the underlying pathogenesis is very complex, receptor pathway may play a major role in it. The receptor for AGEs, namely RAGE, is an immunoglobulin superfamily which can combine with a plurality of ligands. RAGE is expressed in different cell types, including neurons in the whole nervous system. The accumulation of AGEs and the activation of RAGE can lead to oxidative stress, activate NF-κB pathway and up-regulate the target genes expression, trigger inflammation, and result in neuronal cell damage. Animal experiments show that oxidative stress can hinder neurogenesis, increase AGEs production and promote the neuron cell apoptosis (Jing and Zhang, 2011). Therefore, oxidative stress and AGEs accumulation constitute a vicious cycle. There are studies found in STZ-induced diabetic rats that the level of plasma ROS is as high as two times of that in normal rats, which accompanies with AGEs accumulation and RAGE up-regulation; and anti-oxidative stress treatment significantly reduces levels of AGEs and RAGE, thereby prevents neuron damage (Aragno et al., 2005). Toth et al. (2006) reported that knockout of RAGE can dramatically ameliorate neurodegenerative changes in diabetic rats, indicating the significant role of AGEs and RAGE in the development of DCI. Besides RAGE-mediated damage, a recent study reports that the accumulation of AGEs can cause hypertrophy of BBB basement cells, stimulate the production, and secretion of transforming growth factor-β (TGF-β) from the outer membrane, promote the release of vascular endothelial growth factor and MMP-2 from cerebral vascular endothelial cells, and lead to the destruction of BBB (Shimizu et al., 2013).

#### Nitric oxide stress

Nitric oxide (NO) is considered to be a bridge that connects diabetic neuropathy and the organism metabolism. Studies found in DM rats that diabetes will increase nitric oxide synthase (NOS) activity in the brain, and excessive NO will lead to learning and memory dysfunction (Xue et al., 2009; Talarowska et al., 2012). There are three types of NOS, namely neuronal NOS (nNOS), endothelial NOS (eNOS), and inducible NOS (iNOS). The activation of nNOS and eNOS depends on Ca^2+^ and calmodulin (CaM), and small amount of NO can be generated under normal circumstances; iNOS is a non-calcium-dependent enzyme with little or no expression under normal conditions, however, factors including hyperglycemia, AGEs, oxidative stress, ischemia, and hypoxia etc. can activate the enzyme and induce a large amount of NO production. Under physiological conditions, NO can act as a vasodilator and a messenger that mediates information transmission; but at pathological state, as a free radical, it can damage the biomolecules and lead to neurons apoptosis or necrosis (Tokuno et al., 2002; Kim et al., 2010).

There are further studies found that NO stress will influence the synaptic plasticity of the hippocampus. As well known, hippocampus is an important sites that managing learning and memory via long-term potentiation (LTP) induction and maintenance. NO plays an important role in LTP and NOS inhibition can lead to learning and memory dysfunction. Research indicated that sustained hyperglycemia can cause an increase of glutamate, N-methyl-D-aspartate receptor (NMDA) receptor dysfunction, and Ca^2+^/CaM-dependent nNOS activity increase, and the overproduction of NO would result in the enhancement of LTP (Yang et al., 1999; Biessels et al., 2002a). Liu et al. (2003) found that the content and activity of hippocampal NOS is negatively correlated with learning and memory ability in diabetic rats, but this relationship is converse in healthy rats. This can be explained in that excessive NO will hide its LTP enhancing effects by directly inducing neuron damage, thus damaging memory process.

### Inflammation

There is a saying that hyperglycemia is a major cause of DCI, while chronic inflammation can build a bridge between them (Kamboj et al., 2008). Studies have demonstrated that type 2 diabetes is actually a low level chronic inflammatory disease. In Hotamisligil et al. (1993) found in diabetic animals for the first time that the inflammatory cytokines are abnormally increased, and neutralizing inflammatory cytokines by antibodies can increase glucose uptake in peripheral tissues and reduce insulin resistance. Thereafter, amounts of studies found in both animal disease model and diabetic population that type 2 diabetes is accompanied by the elevation of blood lipopolysaccharides (LPS), C-reactive protein (CRP), interleukin (IL)-6, and IL-1, etc. (Yudkin et al., 1999; Cani et al., 2007a; Zhao et al., 2012), further confirmed the low-grade inflammation nature of diabetes. With study progressing, this nature has been recognized to play an important role in the development of DCI. There is a study demonstrated that macrophage activation and infiltration in the nervous system can lead to chronic degenerative disease of the central nervous system (Kierdorf et al., 2010), and blockade the activation of macrophage can significantly decrease levels of malondialdehyde, catalase and superoxide-positive cells in the brain (Wang et al., 2015).

There is currently lack of large cohort study involving inflammatory factors and cognitive impairment risk in diabetic population. A cohort study that included 5,217 cases and followed up for 10 years found that high IL-6 levels in middle age will increase the risk of cognitive decline by as high as 1.81 times (Singh-Manoux et al., 2014), and this was further demonstrated by another study that the negative effects of high IL-6 on cognition will not change concerning use or non-use of statins (Wichmann et al., 2014). TNF-α is closely related with the activity of hippocampus. The increase of TNF-α would specifically damage the spatial memory capacity of animal and decrease the expression of nerve growth factor, thus interference the growth and function of hippocampus (Golan et al., 2004); it was demonstrated in African American patients who have high risk of cardiovascular events that elevated TNF-α dramatically reduces the processing and acting speed of the brain (Windham et al., 2014). Adhesions molecules play an important role in mediating inflammatory cell infiltration and activation. Baydas et al. (2003) reported that the expression of adhesion molecules are significantly increased in the hippocampus of STZ-induced diabetic rats and this elevation is related with memory and learning defects in rats. Study found that the presence of inflammatory cytokines and the activation of NF-κB can directly lead to neuronal dysfunction (Mattson and Camandola, 2001; Liu et al., 2013), and this dysfunction can be relieved by the inhibition of inflammatory signaling pathways (Hofmann et al., 1999).

Besides inflammatory cytokines, amounts of evidences have indicated that metabolic factors also contribute to inflammatory DCI. As discussed above, Rib is elevated in diabetic patients, recent study observed that it can activate RAGE, thereafter activate NF-κB pathway and damage the brain (Han et al., 2014). Meta-analyses have proposed that overweight will increase the risk of cognitive dysfunction (Anstey et al., 2011; Loef and Walach, 2013); in fact, obesity will also induce a low-grade inflammation state and aggravate cognitive impairment in these patients (Nguyen et al., 2014).

A key feature during the inflammatory process is the appearance of Danger Associated Molecular Patterns (DAMP) (Bianchi, 2007). Among DAMPs, a particular molecule that is associated with nerve damage and deserves concern is high mobility group protein box-1 (HMGB-1) (Andersson and Tracey, 2011). HMGB-1 is a nuclear protein that can bind to DNA and regulate gene expression. Abundant evidences suggest that HMGB-1 plays a pivotal role in the tissue repair response, involves in inflammation process, and actively participates in the process of chronic neuropathic disorders (Feldman et al., 2012). Although HMGB-1-related cell signal transduction mechanism is not so clear, it has been recognized that RAGE and TLR2/4 are important receptors mediating its function. Ligand binding studies showed that the affinity of HMGB-1 to bind with RAGE is about 7 times higher than that of AGEs. When HMGB-1 is released into the cytoplasmic, it will exist at all-thiol state (at-HMGB-1) and bind with RAGE to function (Huttunen et al., 2002); there is report suggesting that at-HMGB-1 can also form a complex with CXCL12 and play a role via CXCR4 (Venereau et al., 2013). While in oxidative stress environment, HMGB-1 may experience disulfide reaction and produce the disulfide isoform of HMGB-1 (ds-HMGB-1); ds-HMGB1 mainly participates in the generation of inflammatory cytokine via toll-like receptor 4 (TLR4) (Venereau et al., 2013).

### Glucose and lipid metabolic disorder

High blood glucose is a characteristic of diabetes. The prolonged latency of neuron evoked potentials is a common phenomenon in both type 1 and type 2 diabetes. Study found that the course of diabetes and the level of HbA1c can extend the latency period and insulin therapy can alleviate this change (den Heijer et al., 2003). Moreover, diabetes can also damage the hippocampus structure and result in its dysfunction (Kamal et al., 1999; Gaspar et al., 2010a). A prospective cohort study that included 127,209 people and followed up for 8 years observed that the risk of occurring cognitive impairment in diabetic patients who did not underwent oral hypoglycemic agents treatment will increase to 2.41 times compared with healthy population, while oral hypoglycemic agents treatment can decrease this risk to 1.61 (Hsu et al., 2011). Although hyperglycemia has no effect on the number and concentration of mitochondria and in neurons of hippocampus, it will increase KIF1A, VGluT-1, and synaptotagmin-1 expression, while decrease KIF5B, SNAP 25 and synaptophysin expression (Gaspar et al., 2010b; Baptista et al., 2013), indicating diabetes may have an impact on the neuronal axonal transport at hippocampus.

Diabetic patients are often accompanied with lipid metabolism disorders, manifesting with high cholesterol, high triglycerides, high density lipoprotein and low high-density lipoprotein in the blood. As well known, the brain is an organ rich in cholesterol; however, the brain cholesterol levels are relatively independent of blood cholesterol levels due to the existence of BBB. Although the risk of blood high cholesterol on cognitive impairment is still controversial (Wood et al., 2014), amounts of studies from animal experiments concluded that elevated blood cholesterol levels will promote Aβ amyloid precursor protein production (Posse de Chaves, 2012; Maulik et al., 2013). Brain cholesterol transport between neurons and glial cells is mainly through clusterin/apolipoprotein J and apolipoprotein E (ApoE). However, as ApoE participates in the clearance of Aβ, it is now believed to be involved in the genesis of cognitive impairment. Liao et al. (2014) found that when the Alzheimer's disease mice are treated with ApoE monoclonal antibody, the behavior is improved and accompanies with brain Aβ deposition reduction. In this respect, anti-ApoE antibody may be developed as a potential treatment toward cognitive impairment. Low-density lipoprotein receptor families (LDLR) also play an important role in the pathogenesis of cognitive dysfunction in the brain. Abnormal endocytosis, abnormal lipoproteins signaling pathways and synaptic dysfunction caused by abnormalities of LDLR will impair brain function (Lane-Donovan et al., 2014). In addition, there are a large number of clinical studies have found reduced HDL will increase the risk of cognitive impairment. Therefore, lipid metabolic disorder in diabetic patients plays a role in the pathogenesis of brain cognitive dysfunction.

### Calcium homeostasis imbalance

As well known, calcium (Ca^2+^) homeostasis plays a pivotal role in maintaining the normal function of the organism. It has been well recognized that diabetes and its complications can damage Ca^2+^ homeostasis in neurons, induce degenerative changes of the neuron, and eventually lead to neuronal dysfunction and cell death. Biessels and Gispen (1996) pointed out that ischemia, oxidative stress, and non-enzymatic protein glycation etc. will finally induce Ca^2+^ homeostasis imbalance and lead to nerve degeneration.

Plenty of studies have investigated the involvement of Ca^2+^ homeostasis in DCI. Researchers found that the learning and memory ability of diabetic mice are significantly decreased, and the mRNA and protein expressions of calcium channel protein CaV1.2 in the brain are increased, indicating the synaptic calcium uptake capacity is enhanced; and L-type calcium channel blocker nimodipine can reverse the abnormal expression, improve the Ca^2+^- dependent changes in synaptic plasticity, and ameliorate cognitive dysfunction of the mice (Manschot et al., 2003; Singhal and Sandhir, 2015).

There are many mechanisms that mediate diabetic Ca^2+^ influx, the most important mechanisms lie in the following two pathways: (1) calcium channel excitability enhancement. Calcium channel is commonly activated via the activation of G protein; report demonstrated that the dysfunction of Ca^2+^ channels mediated by G proteins is an important mechanism that leads to Ca^2+^ influx in diabetes (Hall et al., 2001). (2) Ca^2+^-Mg^2+^-ATP enzyme, which is an important regulator that control intracellular Ca^2+^ concentration, the activity of the enzyme in diabetic neuropathy patients is reported to be abnormally enhanced (Migdalis et al., 2000).

The abnormal Ca^2+^ influx would induce Ca^2+^ overloading, and finally result in the apoptosis of the concerned cells. Besides inducing apoptosis process, Ca^2+^ influx can also activate phospholipase, prevent mitochondrial electron transport, release free radicals, and finally lead to cell death (Muranyi et al., 2003).

**Figure 1:**
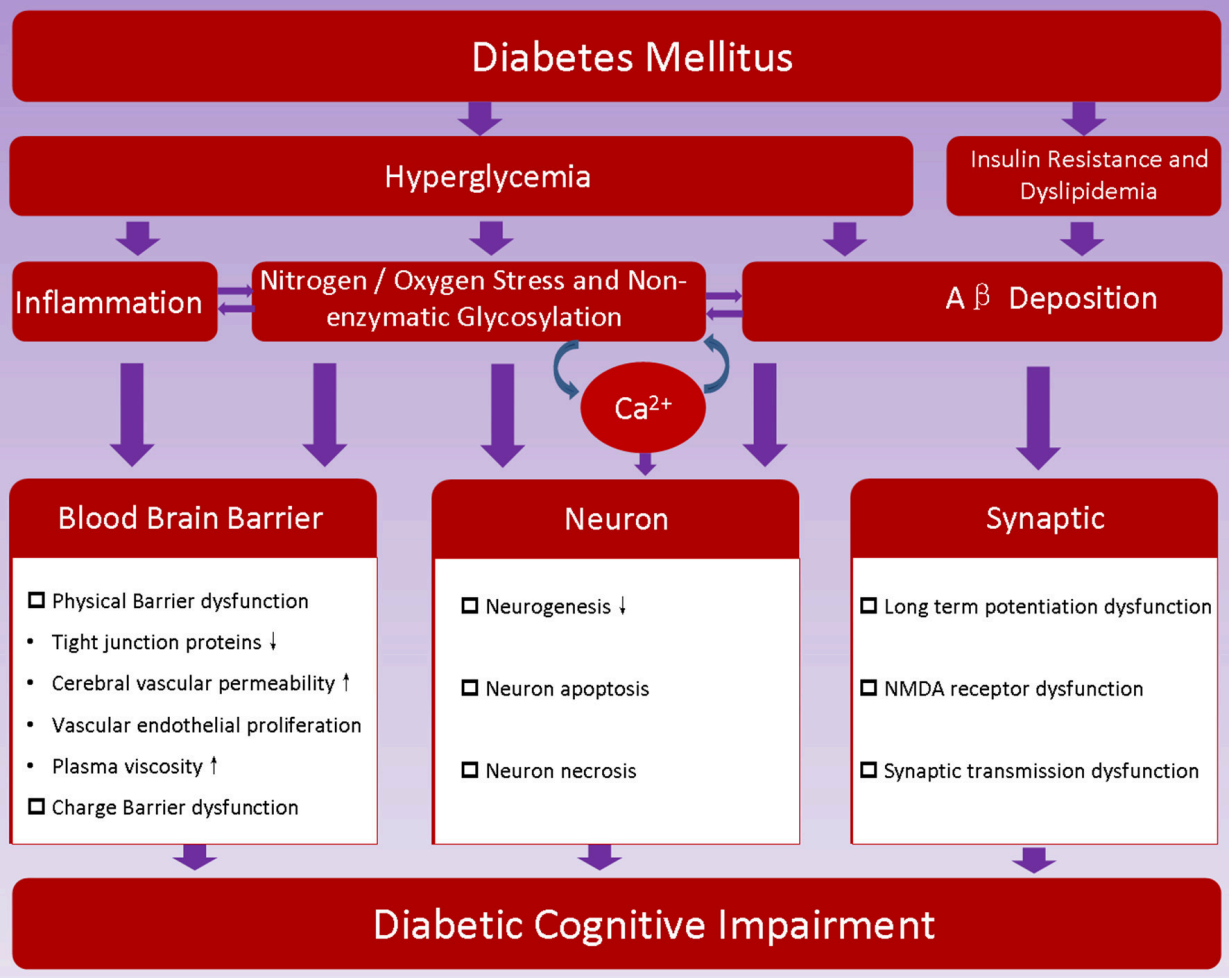
**Proposed pathogenesis of diabetic encephalopathy**. Hyperglycemia, insulin resistance and metabolic disorder are characteristics of diabetes; they may promote dysfunction of blood brain barrier, induce neuron loss, hamper synaptic transmission, and thereafter contribute to the development of diabetic cognitive impairment.

As discussed above, the pathogenesis of DCI is very complex, and its mechanism is not yet entirely clear. At present, it is recognized that the pathogenesis of DCI is closely related with risk factors of diabetes (Figure 1).

## Gut dysbacteriosis contributes to DCI

Microbiota-Gut-brain axis (MGBA) is a two-way adjustment shaft. Amounts of studies have demonstrated that MGBA plays a key role in maintaining the body's metabolism and neuroendocrine stability (Rhee et al., 2009). Although the adjustment factors of MGBA axis is very complex, recent studies indicated that the microbiota and neuroactive peptides are the core, and they may closely related with the development of DCI. Gut dysbacteriosis has been demonstrated to play a role in many psychiatric disorders (Collins et al., 2012; Cryan and Dinan, 2012; Bienenstock et al., 2015).

The specific composition of microbiota is very complex and differs from individuals, but the relative abundance and distribution of the microbiota in healthy population is similar, and the most important two are *Firmicutes* and *Bacteroides*, which account for at least three-quarters of the total microbiota in the organism (Eckburg et al., 2005). The importance of microbiota on health has been recognized in this decade, however, the dialogue pathway and the specific mechanism between gut bacteria and distant organs (such as the brain) has just started. Most recent studies observed that major changes concerning the composition of the microbiota are associated with the occurrence of diabetes (Qin et al., 2012; Forslund et al., 2015), and systematic studies concerning the relationship between microbiota and diabetes have confirmed that gut dysbacteriosis and type 2 diabetes have a direct relationship (Qin et al., 2012; Karlsson et al., 2013), and microbiota may deeply influence the development of diabetes and DCI. Mechanisms including endocrine, immune and neural signaling etc. have been indicated to connect microbiota and brain function (Cryan and Dinan, 2012). Studies observed that germ free mice exhibit a series of spontaneous brain changes including hyperactivity of hypothalamus-pituitary-adrenal axis (Sudo et al., 2004), blood-brain barrier (BBB) permeability increment (Braniste et al., 2014), axon hypermyelination (Hoban et al., 2016), and cognition impairment (Gareau et al., 2011).

### Mass effect of microbiota

The specific mechanism of microbiota imbalance involves diabetes has not been fully elucidated. A study from Cani et al. (2007a) found in mice that high fat diet will increase the amount of toxin-productive Gram-negative bacteria and decrease probiotics (e.g., *Lactobacillus* and *Bifidobacterium*) numbers; on the other hand, stimulating the proliferation of probiotics by adding oligofructose in the food can ameliorate diabetic symptoms (Cani et al., 2007b). In Wu et al. (2010) investigated the stool samples from type 2 diabetic patients and found that the amounts of probiotics is negatively correlated with the level of blood glucose, and hypoglycemic treatment can dramatically increase probiotics level to normal. It is believed that the decrease of gut probiotics will induce the impaired glucose tolerance, reduce sugar-induced insulin secretion, increase the symptoms of endotoxemia, and finally lead to type 2 diabetes (Cani et al., 2007b).

### Co-metabolism products between microbiota and gut play a pivotal role in maintaining the body in a healthy state

**Figure 2:**
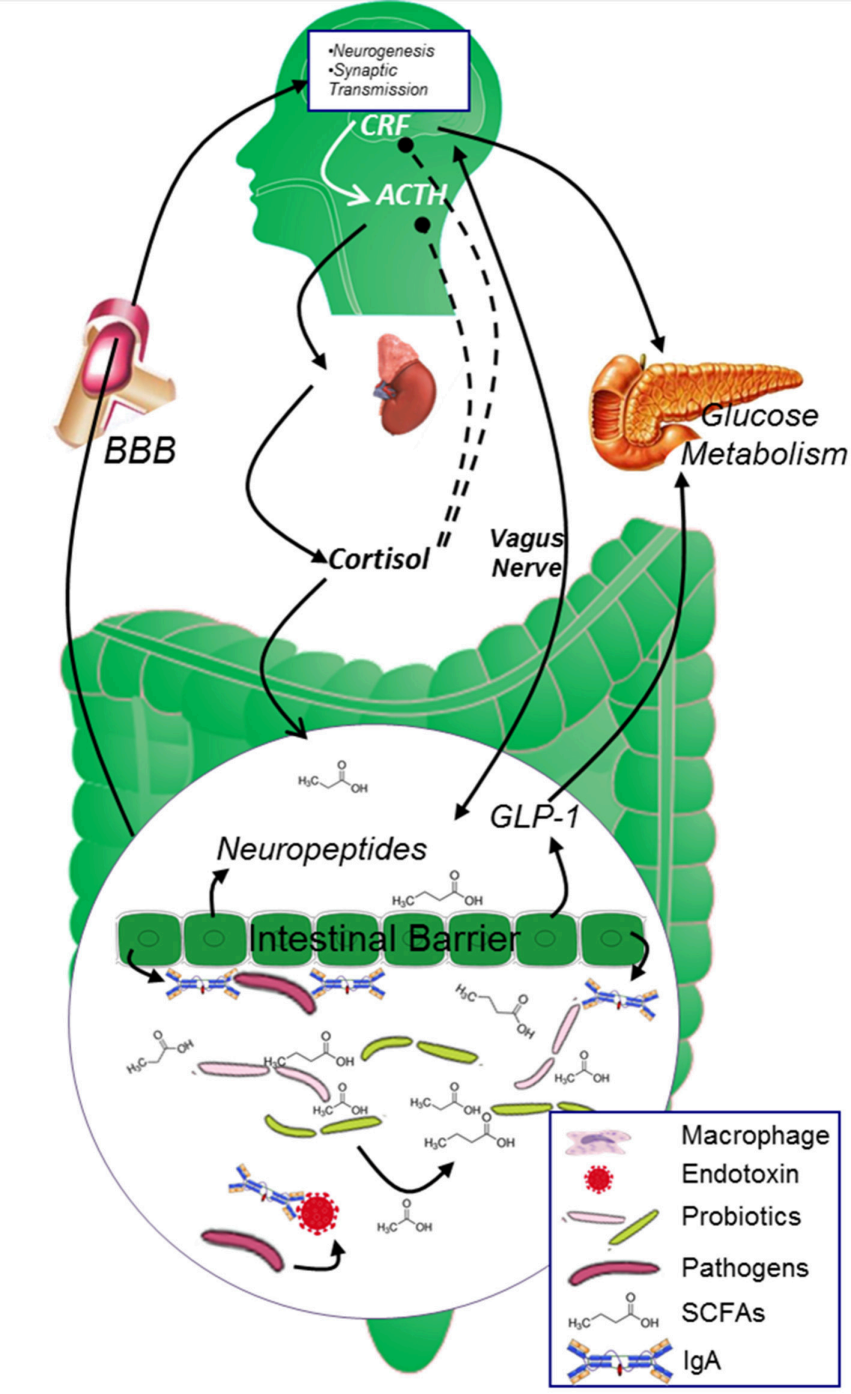
**The two-way cross-talk of MGBA**. In healthy population, the glucose metabolism is strictly controlled by both neuropeptides originated from the brain and endocrine factors secreted from the digestive system. CRF and cortisol modulates the secretion of insulin from islet β cells and maintains blood glucose at reasonable level. Microbiota in the gut digest dietary fiber and “co-metabolize” with gut epithelial cells to keep gut epithelial cell barrier and promote the secretion of neuropeptides, SCFAs originated from Microbiota and neuropeptides generated from the epithelial cells will further circulate to the brain and affect brain function. ACTH, adrenocorticotrophin hormone; AGEs, advanced glycation end products; BBB, blood brain barrier; CRF, corticotropin releasing factor; GABA, γ-aminobutyric acid; ROS, reactive oxygen species; SCFAs, short chain fatty acids.

It is conventionally believed that probiotics may protect the intestinal mucosa against pathogen's attack and guarantee normal intestinal permeability mainly through its “mass effect.” However, recent studies unveiled that the “mass effect” may play only a small role and most of the probiotics' effects are achieved through the “co-metabolism” mechanism between probiotics and the body (Figure 2). Microbiota derived metabolic products have been observed in the blood and in central nervous system, and these products are indicated to be important regulators in gut-brain cross-talk (Leclercq et al., 2017).

The most important metabolic products are small chain fatty acids (SCFAs), including butyric acid, propionic acid, and acetic acid et al. (Miles and Root, 1922; Macfarlane and Macfarlane, 2003). SCFAs are mainly derived from dietary fibers. It is found that SCFAs have neuroactive properties that could directly influence brain function and behavior (MacFabe et al., 2011), promote the development and stability of the nervous system (Stilling et al., 2014) and strengthen the tight connections of intestinal epithelial cells (Reske-Nielsen et al., 1966; Ait-Belgnaoui et al., 2014). Dietary factors can seriously affect the production of gut SCFAs. There are reports demonstrated that rodents fed with western food had reduced levels of SCFAs (Berger et al., 2014; Ojo et al., 2016), compared with controls fed with low-fat diet; and if the body lacks a butyrate-producing bacteria, it is easy to develop type 2 diabetes, while healthy population have more butyrate-producing bacteria than that of diabetic population (Karlsson et al., 2013). More and more studies indicate that SCFAs may influence the body from multiple pathways:

1. Receptor-mediated pathway: SCFAs can regulate the energy balance of the host via the G protein coupled receptor GPR41 and GPR43 (Brown et al., 2003). The combination between SCFAs and GPR41/43 can promote the release of PYY from the intestine, thereby inhibiting intestinal peristalsis and increase SCFAs absorption (Samuel et al., 2008). Another report demonstrated that SCFAs can stimulate GLP-1 secretion via GPR41/43 pathway (Tolhurst et al., 2012), which was shown to help in attenuating pancreatic islet hypertrophy and improving sensitivity (Hwang et al., 2015).
2. Energy source and anti-inflammatory effects: SCFAs can also be utilized by the intestinal epithelial cells as important energy sources, thus affect the epithelial cell proliferation, differentiation and apoptosis. On the other hand, SCFAs also impact functions of dendritic cells function, influences the proliferation of T cell, inhibits NF-κB activation (Hamer et al., 2008; Maslowski et al., 2009; Qin et al., 2012; Trompette et al., 2014; Andrade-Oliveira et al., 2015), therefore show anti-inflammatory property.
3. Neuroprotective effects: SCFAs reach the brain through circulation (Macfabe, 2012) and posses neuroprotective effects (Sun et al., 2015). Reports demonstrate that butyrate, an important component of SCFAs, can improve the age-related memory decline (Reolon et al., 2011) and possess anti-anxiety and anti-depressant effect (Gundersen and Blendy, 2009; Zhu et al., 2009), indicating the positive role of SCFAs in the regulation of central nervous system dysfunction. Studies indicated that SCFAs may influence brain function by regulating neuropeptides secretion. It is found that SCFAs can stimulate the sympathetic nervous system (Kimura et al., 2011), promote the secretion of GABA, serotonin, and dopamine (Grider and Piland, 2007; Lyte, 2011), affect angiogenesis and neurogenesis in the brain (Yoo et al., 2011), influence the cognitive process of learning and memory (Levenson et al., 2004; Li W. et al., 2009; Stefanko et al., 2009) and improve memory performance in the novel object recognition task (Yoo et al., 2015). It is well-known that PYY and glucagon-like peptide type 1 (GLP-1) can not only inhibit intestinal motility and improve glucose metabolism, but also induce satisfy feeling and behavior changes. Researchers found that SCFAs can promote the neuropeptide PYY release from intestinal mucosal epithelial type L cells, and increase GLP-1 and GLP-2 production (Holst, 2007; Samuel et al., 2008; Holzer et al., 2012). Study from Li and colleagues indicated that GLP-1 signaling can promote hippocampal neural plasticity and improve memory function (Li et al., 2012). Besides promoting neuropeptides secretion, SCFAs also play a role in neuron proliferation and differentiation. For instance, SCFAs can directly increase the expression of brain-derived neurotrophic factor (BDNF) and glia-derived neurotrophic factor (GDNF) and inhibit histone deacetylase (HDAC) (Wu et al., 2008).
4. Hippocamp preserving effects: By investigation on the relation between dietary factors and microbiota (Noble et al., 2017), the influence of microbiota on cognition health has been further defined (Fröhlich et al., 2016). Evidences from animal (Kanoski and Davidson, 2011) and human (Baym et al., 2014) have observed the positive relation between SCFAs withdrawn and the impairment of hippocampal-dependent learning and memory function as early as 3 days (Kanoski and Davidson, 2010) after the experiment beginning.

Due to the important role of SCFAs, it has become a hot research topic in the field of diabetes, and some scholars indicated that the co-metabolism between microbiota and the organism may be a potential target for drug treatment and design (Huang et al., 2016; Obrenovich et al., 2016).

### Dysbacteriosis induced endotoxemia leads to inflammatory state of the body and brain

In 2007, Remy Burcelin research group from France firstly proposed that “endotoxemia” originated from intestinal flora is an important factor that triggers inflammatory responses in type 2 diabetes (Cani et al., 2007a). They observed that the composition of intestinal flora in diabetic mice is significantly changed compared with normal mice, beneficial bacteria (e.g., *Bifidobacterium*) which plays a pivotal role in protecting gut barrier is strikingly decreased, the intestinal permeability is increased, and blood toxins (e.g., LPS) are significantly increased (Cani et al., 2007b). Exogenous LPS administration not only lead to weight gain in mice, but also increase the levels of other inflammatory factors in animal (Cani et al., 2007a); and once the animals are treated with antibiotics, with the composition of intestinal flora changes, the intestinal permeability is restored, blood endotoxins are decreased, and the body's inflammatory response is ameliorated (Cani et al., 2008). Although the mechanism of intestinal flora mediated “endotoxemia” is not fully understood, the two interrelated factors may be involved: (1) intestinal dysbacteriosis induced production of endotoxins and inflammatory cytokines, and (2) intestinal dysbacteriosis induced intestinal epithelial permeability increase.

Diabetes has been recognized as a low-grade inflammatory disease, and gut dysbacteriosis will appear under this setting. Inflammatory cells play a pivotal role against gut-derived bacterial products entering the blood circulation. Reports revealed that LPS can stimulate the activation of inflammatory cells (Steenbergen et al., 2015). On the other hand, gut microbiota can also directly stimulate the generation of inflammatory cytokines (Heumann et al., 1994). It is found that intravenous injection of LPS to healthy humans will increase serum levels of inflammatory cytokines and cortisol and decrease memory performance (Krabbe et al., 2005). The findings that inflammatory cytokines are able to reach the brain (Schedlowski et al., 2014) further demonstrate these cytokines may be an important regulator between gut-brain cross-talk. Studies demonstrated that cytokines can increase the expression of serotonin and GABA within the hippocampus (Wang et al., 2012; Jin et al., 2013), thus inhibit brain function. Clinical reports have indicated a positive association between inflammatory cytokines and cognitive decline (Sellbom and Gunstad, 2012). In fact, SCFAs have been demonstrated to inhibit diabetic inflammation. It is found that butyrate has anti-inflammatory actions by preventing LPS-induced activation of inflammatory cells and suppresses nuclear translocation of NF-κB (Segain et al., 2000).

### Dysbacteriosis induced intestinal barrier dysfunction

An important mechanism of microbiota-mediated GBA lies in its effects on intestinal barrier, mainly including the cell barrier and immune barrier. Patients with dysbacteriosis have been observed with higher intestinal permeability, while healthy population without microbial alterations did not (Leclercq et al., 2014), indicating the importance of microbiota in preserving gut barrier.

1. Cell barrier: The cell barrier is mainly composed of enterocytes and tight junctions between neighboring cells (Gallo and Hooper, 2012). Toll like receptors (TLRs) involves in the proliferation and repair process of intestinal epithelial cells (Rakoff-Nahoum et al., 2004). A most recent study reported that microbiota can also affect the activation of TLRs (Caesar et al., 2015), showing the importance of microbiota on the integrity of epithelial cell. LPS and pro-inflammatory cytokines have been demonstrated to down-regulate tight junctions expression and cause disruption of the gut barrier (Al-Sadi et al., 2009, 2014; Guo et al., 2016). The importance of microbiota on protecting the intestinal barrier has been verified in human patients. Previously, a study showed that when the hepatic encephalopathy patients are administrated with oral antibiotics, their brain dysfunction can be ameliorated (Morgan, 1991), indicating antibiotics treatment rebalances the composition and enhances the integrity of intestinal barrier and BBB, thus decreases the permeability of harmful substances across gut epithelial cells and BBB. Recently, there is a research demonstrated that butyrate can stabilize hypoxia-inducible factor (HIF; Kelly et al., 2015), which is critical for preserving gut barrier integrity.
2. Immune barrier: Numerous immune cells in the gut lumen play a key role in defending the body against invading bacteria (Gallo and Hooper, 2012). Studies in sterile animals demonstrated that the microbiota is crucial for the occurrence of gut associated lymphoid tissue (GALT), which plays an pivotal role in the normal secreting of immunoglobulin IgA and effective controlling of the inflammatory response (Quigley, 2008).

### Gut dysbacteriosis increased the permeability of BBB

The direct influence of microbiota on BBB has not yet been fully explored. But findings have indicated the importance of normal gut flora on the stability of BBB. Recently, Braniste et al. (2014) demonstrated that gut microbiota plays an important role in modulating the integrity of BBB; they observed that the permeability of BBB is strikingly increased in germ-free mice, and this is attributed to the down-regulation of tight junction proteins (e.g., occludin and claudin 5) in the brain endothelial cells; once the GF mice are “conventionalized” with flora from pathogen-free mice, the integrity of the BBB is dramatically enhanced, and the authors attributed this to microbiota metabolites, i.e.,: SCFAs. A study from Kanoski et al. (2010) found in rats that low-fiber diet will increase the leaky of BBB in the hippocampus by reducing the tight junction proteins expression; and BBB damage was positively associated with cognitive impairment (Davidson et al., 2012), strongly indicating the importance of microbiota and its metabolic products SCFAs on the integrity of BBB. Besides the effects of SCFAs as discussed above, it is found that SCFAs can also increase the electrical resistance on the epithelial cells and decrease the paracellular permeability (Suzuki et al., 2008); however, the role of SCFAs on ionic charge on BBB still needs to be clarified, the exact mechanisms underlying SCFAs-modulated BBB integrity remains unknown, and study in this area may lead to a new direction toward the treatment of neuro-dysfunction diseases.

### Gut dysbacteriosis related with insulin resistance

Insulin, produced in pancreatic beta cells, plays a central role in modulating blood glucose metabolism, and insulin resistance is one of the characteristic of diabetes. Circulating insulin can cross the BBB and insulin receptors are found to be expressed in synapses (Zhao and Alkon, 2001) and particularly concentrated in the hippocampus (Havrankova et al., 1978). Recent studies revealed insulin signaling in the central nervous system participated in the cognition and neuronal plasticity (Biessels and Reagan, 2015). A research demonstrated that SCFAs deficiency can induce peripheral insulin resistance and cognition impairment in both animals (Gao et al., 2015) and humans (Rönnemaa et al., 2008). The mechanisms are complex. It is found that insulin can activate α-amino-3-hydroxy-5-methyl-4- isoxazolepropionic acid (AMPA) receptors and leads to increased hippocampal long-term potentiation (LTP; Adzovic and Domenici, 2014). Another mechanism involving insulin modulated cognition is via inflammation pathway. Besides possessing peripheral anti-inflammatory effects against endotoxin (Jeschke et al., 2004), recent observation indicated insulin in the central nervous system can attenuate brain inflammation and preserve memory function (Adzovic et al., 2015).

Due to the above important findings, healthy microbiota transfer has been experimentally applied to patients with insulin resistance and received satisfactory outcome (Vrieze et al., 2012). This is demonstrated in low-fiber diet fed rats that antibiotic treatment to these rats helps to improve insulin sensitivity (Suárez-Zamorano et al., 2015) by depleting bad micribiota in the gut.

## Cross-talk between microbiota and HPA axis in the development of DCI

As discussed above, the development of diabetes is dually controlled and regulated by neuroendocrine factors and digestive system. The most important regulatory pathway of human neuroendocrine pathway is hypothalamic-pituitary-adrenal (HPA) axis (Dinan et al., 2006). It was found that HPA is excessively activated in germ-free animal (Sudo et al., 2004). As well known, corticotropin releasing factor (CRF) released from the hypothalamus plays a key role in the HPA axis. More recently, a research reported that diabetes can lead to hyperactivity of the HPA axis and cause functional hypercortisolism (Tirabassi et al., 2016). In fact, CRF has now been believed to be a messenger that modulates microbiota-gut-brain axis (MGBA) (Holzer and Farzi, 2014). Studies found that in germ-free animals the body's CRF and adrenocorticotrophin hormone (ACTH) levels are significantly increased, while brain-derived neurotrophic factor (BDNF) and N-methyl-D-aspartic acid receptor subtypes 2α (NMDAR-2α) are strikingly reduced (Sudo et al., 2004; Crumeyrolle-Arias et al., 2014), indicating microbiota plays an important role in modulating HPA axis and in the process of the neural development. Recent studies indicate hypercortisolism under diabetic state may exacerbate dysbacteriosis-attributed DCI.

CRF includes large family protein peptides, mainly including CRF, Urocortin 1 (UCN1), UCN2, and UCN3, etc. CRF family peptides mainly function through their receptors, namely CRF-R1 and CRF-R2, in which UCN and CRF can simultaneously bind with the two receptor types while UCN3 and UCN2 are highly selective for CRFR2. It has been well studied that CRF family peptides have a wide function on the organism. Recently, studies found that they play an important role in the regulation of diabetes and its complications. For instance, they can inhibit the apoptosis of pancreatic islet cells (Blaabjerg et al., 2014), regulate the release of insulin from islet β cell (van der Meulen et al., 2015), and adjust glucose uptake and utilization of target cells and organs (Chen et al., 2006; Roustit et al., 2014). In the whole animal study, CRF family peptides are found to have a positive effect on diabetes and its complications. Previously, research observed that UCN1 can decrease the content of AGEs in diabetic animals, ameliorate plasma levels of creatinine, and urea nitrogen, reduce the accumulation of glomerular extracellular matrix in the kidney, inhibit the expression of TGF-1β and VEGF, and improve renal injury (Li et al., 2008; Li X. et al., 2009). Recently, it is found that UCN1 can ameliorate diabetic cardiomyopathy via Akt/GSK-3β pathway (Liu et al., 2015). Therefore, converging lines indicate that CRF family peptides play a protective role in the development of diabetes. However, concerning the development of DCI, a depressive conclusion may be drawn, and this conclusion can be traced from the following clues:

### Hypercortisolism contributed microbiota changes

A most recent finding observed that diabetes can lead to hyperactivity of the HPA axis and cause hypercortisolism (Tirabassi et al., 2016). Hypercortisolism can result in a series of dysfunction of or damage to the body, including the composition changes of microbiota and cognitive dysfunction. Evidence from a recently published research showed that the changes of HPA axis in autism patients can lead to a particular structure and composition transform of microbiota, and the extent of this change is closely related to the severity of the disease (Mayer et al., 2014). Another study showed that 2 h of the environmental stress can change the microbiota (Galley et al., 2014), indicating the important function of CRF family peptides on the microbiota. On the other hand, diabetic dysbacteriosis leads to the dysfunction of intestinal barrier and BBB and promotes the development of DCI, hypercortisolism should obviously facilitate effects of dysbacteriosis on DCI.

### Hypercortisolism aggravates intestinal barrier abnormal

As discussed above, the intestine and microbiota play an important role in the development of diabetes. CRF is a neuropeptide which is closely related to the initiation of stress. Studies found that the activation of peripheral CRF receptors may lead to stress-associated physiological function changes of the intestine (Kiank et al., 2010). Amounts of evidences indicate that the integrity of the intestinal epithelium, intestinal mucosal immune system, and microbiota composition can be affected by the CRF family peptides. The activation of CRF receptors can directly cause the damage of intestinal barrier function (Söderholm et al., 2002), increase the trans-intestinal epithelium ability of the bacteria, and promote the infiltration of inflammatory cells in the intestinal lamina (Rhee et al., 2009). Previous study also observed that the activation of CRF receptors can increase the permeability of the blood vessels and induce tissue edema via the activating mast cells and histamine H1 receptor (Wu et al., 2006).

### Hypercortisolism leads to the secretion of inhibitory neurotransmitter

The balance between excitatory and inhibitory neurotransmitters is very important in maintaining the normal function of brain. Studies found that hypercortisolism induced imbalance of microbiota may promote the abnormal secretion of neurotransmitter such as serotonin (also known as 5-HT) (Diaz Heijtz et al., 2011), γ-aminobutyric acid (GABA), and histamine (Saulnier et al., 2013) et al., and finally results in anxiety symptoms.

Study concerning the role and mechanism of HPA on DCI is rare. Evidence from existed references depicts us with an image that the involvement of HPA on DCI is very complex: it is beneficial to blood metabolism on the one hand, and hypercortisolism is harmful to brain function on the other hand. Therefore, strictly controlling the stable state of HPA is very important, and study concerning this area may bring us with new understandings of DCI.

**Figure 3:**
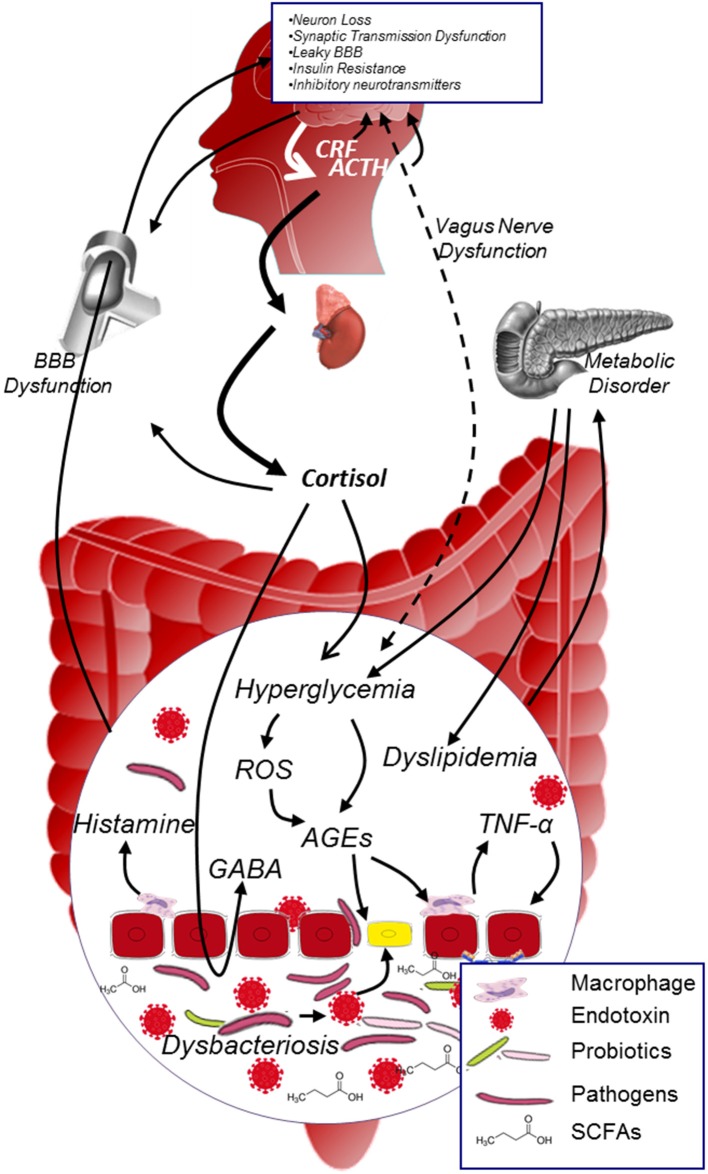
**MGBA modulated DCI**. In diabetic population, metabolic disorder and hypercortisolism will exacerbate hyperglycemia, promote the generation of AGEs and inflammatory cytokines in the internal environment; on the other hand, dysbacteriosis in the gut will dramatically increase the production of endotoxin and decrease the level of SCFAs. Both dysbacteriosis and internal environment disorder will lead to the intestinal barrier and BBB dysfunction, facilitate harmful substance (e.g., AGEs, endotoxin, pathogens etc.) to access to neurons, and thus contribute to the development of DCI. ACTH, adrenocorticotrophin hormone; AGEs, advanced glycation end products; BBB, blood brain barrier; CRF, corticotropin releasing factor; GABA, γ-aminobutyric acid; ROS, reactive oxygen species; SCFAs, short chain fatty acids.

Converging lines have suggested that MGBA plays a role in modulating DCI (Figure 3). On the one hand, microbiota possess various effects on protecting the organism: it enhances the integrity of the gut epithelial cells, limits the low grade inflammation in many chronic disease, improves the glucose metabolism, reduces the oxidative stress and AGEs production, mediates the secretion of neuropeptides, influences the neurogenesis, and affects the activation of HPA. On the other hand, HPA plays a key role in regulating the neuroendocrine pathway: it interacts with microbiota (although the specific mechanism deserves to be elucidated), participates in the stress process, regulates glucose metabolism, and affects the permeability of blood vessels etc. The interaction between microbiota and HPA is complex, the balance between these two factors is pivotal in maintaining normal function of the body. Investigating microbiota-HPA mediated MGBA should have great impact on understanding the development of DCI.

## Nerve connection between gut and brain

Vagus nerve, which is widely distributed in the gut, has also been well-established as a connection among microbiota, the peripheral nervous system, and the central nervous system (Layé et al., 1995; Forsythe and Kunze, 2013). It is observed that gut microbiota can directly activate the enteric nervous system (Furness, 2012) and further transmit information into brain via vagus nerve. Besides this “by-pass” pathway, vagus nerve endings also express receptors for inflammatory cytokines including IL-1 and prostaglandins (Ek et al., 1998), therefore can be directly activated by gut-derived inflammatory cytokines. However, studies concerning this area are relatively few, and further investigations deserve to be carried out to unveil the direct connection between microbiota and brain in this aspect of view.

## Conclusion

With an aging population worldwide, the incidence of diabetes and diabetic complications has dramatically increased. One of an important complication accompanied with diabetes is cognitive impairment. Evidences within this decade strongly suggest that risk factors such as oxidative stress, inflammation, and AGEs, which accounts for the development of diabetes, may also seriously affect brain function. The most recent studies concerning gut microbiota elucidated one more possible mechanism on the progression of DCI. From the discussions above, we may predict that intervention targets such as reducing ROS and AGEs generation, inhibiting over-inflammation impairment or re-balancing gut-sourced health-preserving effects etc. should help to delay the development of DCI. In view of irreversible development of diabetes, early intervention and therapeutic strategies targeting the gut microbiota may be effective on reducing or ameliorating the progression of DCI.

## Author contributions

All authors listed, have made substantial, direct and intellectual contribution to the work, and approved it for publication.

## Funding

This work was supported by Science and Technology Development Fund of Macau (FDCT: 071/2014/A).

### Conflict of interest statement

The authors declare that the research was conducted in the absence of any commercial or financial relationships that could be construed as a potential conflict of interest. The reviewer YX declares to co-supervise a student with one of the authors YX and the absence of any scientific collaboration to the handling Editor, who ensured that the process met the standards of a fair and objective review.

## References

[B1] Abbott N. J. Rönnbäck L. Hansson E. (2006). Astrocyte-endothelial interactions at the blood-brain barrier. Nat. Rev. Neurosci. 7, 41–53. 10.1038/nrn1824 16371949

[B2] Adzovic L. Domenici L. (2014). Insulin induces phosphorylation of the AMPA receptor subunit GluR1, reversed by ZIP, and over-expression of Protein Kinase M zeta, reversed by amyloid beta. J. Neurochem. 131, 582–587. 10.1111/jnc.12947 25230927

[B3] Adzovic L. Lynn A. E. D'Angelo H. M. Crockett A. M. Kaercher R. M. Royer S. E.. (2015). Insulin improves memory and reduces chronic neuroinflammation in the hippocampus of young but not aged brains. J. Neuroinflammation 12:63. 10.1186/s12974-015-0282-z 25889938 PMC4391678

[B4] Ait-Belgnaoui A. Colom A. Braniste V. Ramalho L. Marrot A. Cartier C.. (2014). Probiotic gut effect prevents the chronic psychological stress-induced brain activity abnormality in mice. Neurogastroenterol. Motil. 26, 510–520. 10.1111/nmo.12295 24372793

[B5] Al-Sadi R. Boivin M. Ma T. (2009). Mechanism of cytokine modulation of epithelial tight junction barrier. Front. Biosci. 14, 2765–2778. 10.2741/3413 19273235 PMC3724223

[B6] Al-Sadi R. Ye D. Boivin M. Guo S. Hashimi M. Ereifej L.. (2014). Interleukin-6 modulation of intestinal epithelial tight junction permeability is mediated by JNK pathway activation of claudin-2 gene. PLoS ONE 9:e85345. 10.1371/journal.pone.0085345 24662742 PMC3963839

[B7] Alvarez E. O. Beauquis J. Revsin Y. Banzan A. M. Roig P. De Nicola A. F.. (2009). Cognitive dysfunction and hippocampal changes in experimental type 1 diabetes. Behav. Brain Res. 198, 224–230. 10.1016/j.bbr.2008.11.001 19041902

[B8] Andersson U. Tracey K. J. (2011). HMGB1 is a therapeutic target for sterile inflammation and infection. Annu. Rev. Immunol. 29, 139–162. 10.1146/annurev-immunol-030409-101323 21219181 PMC4536551

[B9] Andrade-Oliveira V. Amano M. T. Correa-Costa M. Castoldi A. Felizardo R. J. de Almeida D. C.. (2015). Gut bacteria products prevent AKI Induced by ischemia-reperfusion. J. Am. Soc. Nephrol. 26, 1877–1888. 10.1681/ASN.2014030288 25589612 PMC4520159

[B10] Anstey K. J. Cherbuin N. Budge M. Young J. (2011). Body mass index in midlife and late-life as a risk factor for dementia: a meta-analysis of prospective studies. Obes. Rev. 12, e426–e437. 10.1111/j.1467-789x.2010.00825.x 21348917

[B11] Aragno M. Mastrocola R. Medana C. Restivo F. Catalano M. G. Pons N.. (2005). Up-regulation of advanced glycated products receptors in the brain of diabetic rats is prevented by antioxidant treatment. Endocrinology 146, 5561–5567. 10.1210/en.2005-0712 16166220

[B12] Baptista F. I. Pinto M. J. Elvas F. Almeida R. D. Ambrósio A. F. (2013). Diabetes alters KIF1A and KIF5B motor proteins in the hippocampus. PLoS ONE 8:e65515. 10.1371/journal.pone.0065515 23776493 PMC3680435

[B13] Baydas G. Nedzvetskii V. S. Nerush P. A. Kirichenko S. V. Yoldas T. (2003). Altered expression of NCAM in hippocampus and cortex may underlie memory and learning deficits in rats with streptozotocin-induced diabetes mellitus. Life Sci. 73, 1907–1916. 10.1016/S0024-3205(03)00561-7 12899916

[B14] Baym C. L. Khan N. A. Monti J. M. Raine L. B. Drollette E. S. Moore R. D.. (2014). Dietary lipids are differentially associated with hippocampal-dependent relational memory in prepubescent children. Am. J. Clin. Nutr. 99, 1026–1032. 10.3945/ajcn.113.079624 24522447 PMC3985209

[B15] Bercik P. Denou E. Collins J. Jackson W. Lu J. Jury J.. (2011). The intestinal microbiota affect central levels of brain-derived neurotropic factor and behavior in mice. Gastroenterology 141, 599–609, 609.e1–3. 10.1053/j.gastro.2011.04.052 21683077

[B16] Berger K. Falck P. Linninge C. Nilsson U. Axling U. Grey C.. (2014). Cereal byproducts have prebiotic potential in mice fed a high-fat diet. J. Agric. Food. Chem. 62, 8169–8178. 10.1021/jf502343v 25041844

[B17] Bianchi M. E. (2007). DAMPs, PAMPs and alarmins: all we need to know about danger. J. Leukoc. Biol. 81, 1–5. 10.1189/jlb.0306164 17032697

[B18] Bienenstock J. Kunze W. Forsythe P. (2015). Microbiota and the gut-brain axis. Nutr. Rev. 73(Suppl. 1), 28–31. 10.1093/nutrit/nuv019 26175487

[B19] Biessels G. Gispen W. H. (1996). The calcium hypothesis of brain aging and neurodegenerative disorders: significance in diabetic neuropathy. Life Sci. 59, 379–387. 10.1016/0024-3205(96)00316-5 8761325

[B20] Biessels G. J. Deary I. J. Ryan C. M. (2008). Cognition and diabetes: a lifespan perspective. Lancet Neurol. 7, 184–190. 10.1016/S1474-4422(08)70021-8 18207116

[B21] Biessels G. J. van der Heide L. P. Kamal A. Bleys R. L. Gispen W. H. (2002a). Ageing and diabetes: implications for brain function. Eur. J. Pharmacol. 441, 1–14. 10.1016/S0014-2999(02)01486-3 12007915

[B22] Biessels G. J. ter Laak M. P. Hamers F. P. Gispen W. H. (2002b). Neuronal Ca^2+^ disregulation in diabetes mellitus. Eur. J. Pharmacol. 447, 201–209. 10.1016/S0014-2999(02)01844-7 12151012

[B23] Biessels G. J. Reagan L. P. (2015). Hippocampal insulin resistance and cognitive dysfunction. Nat. Rev. Neurosci. 16, 660–671. 10.1038/nrn4019 26462756

[B24] Blaabjerg L. Christensen G. L. Matsumoto M. van der Meulen T. Huising M. O. Billestrup N.. (2014). CRFR1 activation protects against cytokine-induced β-cell death. J. Mol. Endocrinol. 53, 417–427. 10.1530/JME-14-0056 25324488 PMC4518718

[B25] Brands A. M. Biessels G. J. Kappelle L. J. de Haan E. H. de Valk H. W. Algra A.. (2007). Cognitive functioning and brain MRI in patients with type 1 and type 2 diabetes mellitus: a comparative study. Dement. Geriatr. Cogn. Disord. 23, 343–350. 10.1159/000100980 17374953

[B26] Braniste V. Al-Asmakh M. Kowal C. Anuar F. Abbaspour A. Tóth M. Korecka A.. (2014). The gut microbiota influences blood-brain barrier permeability in mice. Sci. Transl. Med. 6, 263ra158. 10.1126/scitranslmed.3009759 25411471 PMC4396848

[B27] Briani C. Ruggero S. Alaedini A. Nardelli E. Ferrari S. Wirguin I.. (2002). Anti-heparan sulfate antibodies in neurological disease. Muscle Nerve 26, 713–715. 10.1002/mus.10226 12402295

[B28] Brown A. J. Goldsworthy S. M. Barnes A. A. Eilert M. M. Tcheang L. Daniels D.. (2003). The Orphan G protein-coupled receptors GPR41 and GPR43 are activated by propionate and other short chain carboxylic acids. J. Biol. Chem. 278, 11312–11319. 10.1074/jbc.M211609200 12496283

[B29] Brownlee M. Vlassara H. Cerami A. (1984). Nonenzymatic glycosylation and the pathogenesis of diabetic complications. Ann. Intern. Med. 101, 527–537. 10.7326/0003-4819-101-4-527 6383165

[B30] Caesar R. Tremaroli V. Kovatcheva-Datchary P. Cani P. D. Bäckhed F. (2015). Crosstalk between gut microbiota and dietary lipids aggravates WAT Inflammation through TLR Signaling. Cell Metab. 22, 658–668. 10.1016/j.cmet.2015.07.026 26321659 PMC4598654

[B31] Cani P. D. Amar J. Iglesias M. A. Poggi M. Knauf C. Bastelica D.. (2007a). Metabolic endotoxemia initiates obesity and insulin resistance. Diabetes 56, 1761–1772. 10.2337/db06-1491 17456850

[B32] Cani P. D. Bibiloni R. Knauf C. Waget A. Neyrinck A. M. Delzenne N. M.. (2008). Changes in gut microbiota control metabolic endotoxemia-induced inflammation in high-fat diet-induced obesity and diabetes in mice. Diabetes 57, 1470–1481. 10.2337/db07-1403 18305141

[B33] Cani P. D. Neyrinck A. M. Fava F. Knauf C. Burcelin R. G. Tuohy K. M.. (2007b). Selective increases of bifidobacteria in gut microflora improve high-fat-diet-induced diabetes in mice through a mechanism associated with endotoxaemia. Diabetologia 50, 2374–2383. 10.1007/s00125-007-0791-0 17823788

[B34] Cardoso S. Correia S. C. Santos R. X. Carvalho C. Candeias E. Duarte A. I.. (2013). Hyperglycemia, hypoglycemia and dementia: role of mitochondria and uncoupling proteins. Curr. Mol. Med. 13, 586–601. 10.2174/1566524011313040010 22934852

[B35] Chang M. (2016). Restructuring of the extracellular matrix in diabetic wounds and healing: a perspective. Pharmacol. Res. 107, 243–248. 10.1016/j.phrs.2016.03.008 27033051

[B36] Chen A. Brar B. Choi C. S. Rousso D. Vaughan J. Kuperman Y.. (2006). Urocortin 2 modulates glucose utilization and insulin sensitivity in skeletal muscle. Proc. Natl. Acad. Sci. U.S.A. 103, 16580–16585. 10.1073/pnas.0607337103 17050686 PMC1637624

[B37] Collins S. M. Surette M. Bercik P. (2012). The interplay between the intestinal microbiota and the brain. Nat. Rev. Microbiol. 10, 735–742. 10.1038/nrmicro2876 23000955

[B38] Crumeyrolle-Arias M. Jaglin M. Bruneau A. Vancassel S. Cardona A. Daugé V.. (2014). Absence of the gut microbiota enhances anxiety-like behavior and neuroendocrine response to acute stress in rats. Psychoneuroendocrinology 42, 207–217. 10.1016/j.psyneuen.2014.01.014 24636517

[B39] Cryan J. F. Dinan T. G. (2012). Mind-altering microorganisms: the impact of the gut microbiota on brain and behaviour. Nat. Rev. Neurosci. 13, 701–712. 10.1038/nrn3346 22968153

[B40] Dahl-Jørgensen K. Brinchmann-Hansen O. Hanssen K. F. Ganes T. Kierulf P. Smeland E.. (1986). Effect of near normoglycaemia for two years on progression of early diabetic retinopathy, nephropathy, and neuropathy: the Oslo study. Br. Med. J. (Clin. Res. Ed.) 293, 1195–1199. 10.1136/bmj.293.6556.1195 3096429 PMC1341978

[B41] Dai J. Vrensen G. F. Schlingemann R. O. (2002). Blood-brain barrier integrity is unaltered in human brain cortex with diabetes mellitus. Brain Res. 954, 311–316. 10.1016/S0006-8993(02)03294-8 12414115

[B42] Dalal P. M. Parab P. V. (2002). Cerebrovascular disease in type 2 diabetes mellitus. Neurol. India 50, 380–385. 12577084

[B43] Davidson T. L. Monnot A. Neal A. U. Martin A. A. Horton J. J. Zheng W. (2012). The effects of a high-energy diet on hippocampal-dependent discrimination performance and blood-brain barrier integrity differ for diet-induced obese and diet-resistant rats. Physiol. Behav. 107, 26–33. 10.1016/j.physbeh.2012.05.015 22634281 PMC3409296

[B44] den Heijer T. Vermeer S. E. van Dijk E. J. Prins N. D. Koudstaal P. J. Hofman A.. (2003). Type 2 diabetes and atrophy of medial temporal lobe structures on brain MRI. Diabetologia 46, 1604–1610. 10.1007/s00125-003-1235-0 14595538

[B45] Diaz Heijtz R. Wang S. Anuar F. Qian Y. Björkholm B. Samuelsson A.. (2011). Normal gut microbiota modulates brain development and behavior. Proc. Natl. Acad. Sci. U.S.A. 108, 3047–3052. 10.1073/pnas.1010529108 21282636 PMC3041077

[B46] Dinan T. G. Cryan J. F. (2012). Regulation of the stress response by the gut microbiota: implications for psychoneuroendocrinology. Psychoneuroendocrinology 37, 1369–1378. 10.1016/j.psyneuen.2012.03.007 22483040

[B47] Dinan T. G. Quigley E. M. Ahmed S. M. Scully P. O'Brien S. O'Mahony L.. (2006). Hypothalamic-pituitary-gut axis dysregulation in irritable bowel syndrome: plasma cytokines as a potential biomarker? Gastroenterology 130, 304–311. 10.1053/j.gastro.2005.11.033 16472586

[B48] Duran-Jimenez B. Dobler D. Moffatt S. Rabbani N. Streuli C. H. Thornalley P. J.. (2009). Advanced glycation end products in extracellular matrix proteins contribute to the failure of sensory nerve regeneration in diabetes. Diabetes 58, 2893–2903. 10.2337/db09-0320 19720799 PMC2780874

[B49] Eckburg P. B. Bik E. M. Bernstein C. N. Purdom E. Dethlefsen L. Sargent M.. (2005). Diversity of the human intestinal microbial flora. Science 308, 1635–1638. 10.1126/science.1110591 15831718 PMC1395357

[B50] Ek M. Kurosawa M. Lundeberg T. Ericsson A. (1998). Activation of vagal afferents after intravenous injection of interleukin-1beta: role of endogenous prostaglandins. J. Neurosci. 18, 9471–9479. 9801384 10.1523/JNEUROSCI.18-22-09471.1998 PMC6792875

[B51] Feldman P. Due M. R. Ripsch M. S. Khanna R. White F. A. (2012). The persistent release of HMGB1 contributes to tactile hyperalgesia in a rodent model of neuropathic pain. J. Neuroinflammation 9:180. 10.1186/1742-2094-9-180 22824385 PMC3488576

[B52] Forslund K. Hildebrand F. Nielsen T. Falony G. Le Chatelier E. Sunagawa S.. (2015). Disentangling type 2 diabetes and metformin treatment signatures in the human gut microbiota. Nature 528, 262–266. 10.1038/nature15766 26633628 PMC4681099

[B53] Forsythe P. Kunze W. A. (2013). Voices from within: gut microbes and the CNS. Cell. Mol. Life. Sci. 70, 55–69. 10.1007/s00018-012-1028-z 22638926 PMC11113561

[B54] Foster J. A. McVey Neufeld K. A. (2013). Gut-brain axis: how the microbrome influences anxiety and depression. Trends Neurosci. 36, 305–312. 10.1016/j.tins.2013.01.005 23384445

[B55] Fouyas I. P. Kelly P. A. Ritchie I. M. Lammie G. A. Whittle I. R. (2003). Cerebrovascular responses to pathophysiological insult in diabetic rats. J. Clin. Neurosci. 10, 88–91. 10.1016/S0967-5868(02)00247-3 12464531

[B56] Fröhlich E. E. Farzi A. Mayerhofer R. Reichmann F. Jačan A. Wagner B.. (2016). Cognitive impairment by antibiotic-induced gut dysbiosis: analysis of gut microbiota-brain communication. Brain Behav. Immun. 56, 140–155. 10.1016/j.bbi.2016.02.020 26923630 PMC5014122

[B57] Furness J. B. (2012). The enteric nervous system and neurogastroenterology. Nat. Rev. Gastroenterol. Hepatol. 9, 286–294. 10.1038/nrgastro.2012.32 22392290

[B58] Galley J. D. Nelson M. C. Yu Z. Dowd S. E. Walter J. Kumar P. S.. (2014). Exposure to a social stressor disrupts the community structure of the colonic mucosa-associated microbiota. BMC Microbiol. 14:189. 10.1186/1471-2180-14-189 25028050 PMC4105248

[B59] Gallo R. L. Hooper L. V. (2012). Epithelial antimicrobial defence of the skin and intestine. Nat. Rev. Immunol. 12, 503–516. 10.1038/nri3228 22728527 PMC3563335

[B60] Gao Y. Xiao Y. Miao R. Zhao J. Zhang W. Huang G.. (2015). The characteristic of cognitive function in Type 2 diabetes mellitus. Diabetes Res. Clin. Pract. 109, 299–305. 10.1016/j.diabres.2015.05.019 26004430

[B61] Gareau M. G. Wine E. Rodrigues D. M. Cho J. H. Whary M. T. Philpott D. J.. (2011). Bacterial infection causes stress-induced memory dysfunction in mice. Gut 60, 307–317. 10.1136/gut.2009.202515 20966022

[B62] Garro A. Chodobski A. Szmydynger-Chodobska J. Shan R. Bialo S. R. Bennett J.. (2017). Circulating matrix metalloproteinases in children with diabetic ketoacidosis. Pediatr. Diabetes 18, 95–102. 10.1111/pedi.12359 26843101 PMC4974171

[B63] Garsen M. Rops A. L. Rabelink T. J. Berden J. H. van der Vlag J. (2014). The role of heparanase and the endothelial glycocalyx in the development of proteinuria. Nephrol. Dial. Transplant. 29, 49–55. 10.1093/ndt/gft410 24166469

[B64] Gaspar J. M. Baptista F. I. Galvão J. Castilho A. F. Cunha R. A. Ambrósio A. F. (2010a). Diabetes differentially affects the content of exocytotic proteins in hippocampal and retinal nerve terminals. Neuroscience 169, 1589–1600. 10.1016/j.neuroscience.2010.06.021 20600668

[B65] Gaspar J. M. Castilho Á. Baptista F. I. Liberal J. Ambrósio A. F. (2010b). Long-term exposure to high glucose induces changes in the content and distribution of some exocytotic proteins in cultured hippocampal neurons. Neuroscience 171, 981–992. 10.1016/j.neuroscience.2010.10.019 20950673

[B66] Giannini C. Dyck P. J. (1995). Basement membrane reduplication and pericyte degeneration precede development of diabetic polyneuropathy and are associated with its severity. Ann. Neurol. 37, 498–504. 10.1002/ana.410370412 7717686

[B67] Golan H. Levav T. Mendelsohn A. Huleihel M. (2004). Involvement of tumor necrosis factor alpha in hippocampal development and function. Cereb. Cortex 14, 97–105. 10.1093/cercor/bhg108 14654461

[B68] Grider J. R. Piland B. E. (2007). The peristaltic reflex induced by short-chain fatty acids is mediated by sequential release of 5-HT and neuronal CGRP but not BDNF. Am. J. Physiol. Gastrointest. Liver Physiol. 292, G429–G437. 10.1152/ajpgi.00376.2006 16973914

[B69] Grillo C. A. Piroli G. G. Rosell D. R. Hoskin E. K. Mcewen B. S. Reagan L. P. (2003). Region specific increases in oxidative stress and superoxide dismutase in the hippocampus of diabetic rats subjected to stress. Neuroscience 121, 133–140. 10.1016/S0306-4522(03)00343-9 12946706

[B70] Gudala K. Bansal D. Schifano F. Bhansali A. (2013). Diabetes mellitus and risk of dementia: a meta-analysis of prospective observational studies. J. Diabetes Investig. 4, 640–650. 10.1111/jdi.12087 24843720 PMC4020261

[B71] Gundersen B. B. Blendy J. A. (2009). Effects of the histone deacetylase inhibitor sodium butyrate in models of depression and anxiety. Neuropharmacology 57, 67–74. 10.1016/j.neuropharm.2009.04.008 19393671 PMC2702471

[B72] Guo H. Xu Y. Huang W. Zhou H. Zheng Z. Zhao Y.. (2016). Kuwanon G preserves LPS-induced disruption of gut epithelial barrier *in vitro*. Molecules 21:E1597. 10.3390/molecules21111597 27879681 PMC6272946

[B73] Hall K. E. Liu J. Sima A. A. Wiley J. W. (2001). Impaired inhibitory G-protein function contributes to increased calcium currents in rats with diabetic neuropathy. J. Neurophysiol. 86, 760–770. 11495948 10.1152/jn.2001.86.2.760

[B74] Hamer H. M. Jonkers D. Venema K. Vanhoutvin S. Troost F. J. Brummer R. J. (2008). Review article: the role of butyrate on colonic function. Aliment. Pharmacol. Ther. 27, 104–119. 10.1111/j.1365-2036.2007.03562.x 17973645

[B75] Han C. Lu Y. Wei Y. Liu Y. He R. (2011). D-ribose induces cellular protein glycation and impairs mouse spatial cognition. PLoS ONE 6:e24623. 10.1371/journal.pone.0024623 21966363 PMC3169629

[B76] Han C. Lu Y. Wei Y. Wu B. Liu Y. He R. (2014). D-ribosylation induces cognitive impairment through RAGE-dependent astrocytic inflammation. Cell Death Dis. 5:e1117. 10.1038/cddis.2014.89 24625976 PMC3973213

[B77] Havrankova J. Schmechel D. Roth J. Brownstein M. (1978). Identification of insulin in rat brain. Proc. Natl. Acad. Sci. U.S.A. 75, 5737–5741. 10.1073/pnas.75.11.5737 364489 PMC393044

[B78] Hawkins B. T. Lundeen T. F. Norwood K. M. Brooks H. L. Egleton R. D. (2007). Increased blood-brain barrier permeability and altered tight junctions in experimental diabetes in the rat: contribution of hyperglycaemia and matrix metalloproteinases. Diabetologia 50, 202–211. 10.1007/s00125-006-0485-z 17143608

[B79] Heumann D. Barras C. Severin A. Glauser M. P. Tomasz A. (1994). Gram-positive cell walls stimulate synthesis of tumor necrosis factor alpha and interleukin-6 by human monocytes. Infect. Immun. 62, 2715–2721. 7516310 10.1128/iai.62.7.2715-2721.1994 PMC302873

[B80] Hoban A. E. Stilling R. M. Ryan F. J. Shanahan F. Dinan T. G. Claesson M. J.. (2016). Regulation of prefrontal cortex myelination by the microbiota. Transl. Psychiatry 6:e774. 10.1038/tp.2016.42 27045844 PMC4872400

[B81] Hofmann M. A. Schiekofer S. Isermann B. Kanitz M. Henkels M. Joswig M.. (1999). Peripheral blood mononuclear cells isolated from patients with diabetic nephropathy show increased activation of the oxidative-stress sensitive transcription factor NF-κB. Diabetologia 42, 222–232. 10.1007/s001250051142 10064103

[B82] Holst J. J. (2007). The physiology of glucagon-like peptide 1. Physiol. Rev. 87, 1409–1439. 10.1152/physrev.00034.2006 17928588

[B83] Holzer P. Farzi A. (2014). Neuropeptides and the microbiota-gut-brain axis. Adv. Exp. Med. Biol. 817, 195–219. 10.1007/978-1-4939-0897-4_9 24997035 PMC4359909

[B84] Holzer P. Reichmann F. Farzi A. (2012). Neuropeptide Y, peptide YY and pancreatic polypeptide in the gut-brain axis. Neuropeptides 46, 261–274. 10.1016/j.npep.2012.08.005 22979996 PMC3516703

[B85] Hotamisligil G. S. Shargill N. S. Spiegelman B. M. (1993). Adipose expression of tumor necrosis factor-alpha: direct role in obesity-linked insulin resistance. Science 259, 87–91. 10.1126/science.7678183 7678183

[B86] Hsu C. C. Wahlqvist M. L. Lee M. S. Tsai H. N. (2011). Incidence of dementia is increased in type 2 diabetes and reduced by the use of sulfonylureas and metformin. J. Alzheimers Dis. 24, 485–493. 10.3233/JAD-2011-101524 21297276

[B87] Huang W. Zhou L. Guo H. Xu Y. Xu Y. (2016). The role of short-chain fatty acids in kidney injury induced by gut-derived inflammatory response. Metabolism 68, 20–30. 10.1016/j.metabol.2016.11.006 28183450

[B88] Huttunen H. J. Fages C. Kuja-Panula J. Ridley A. J. Rauvala H. (2002). Receptor for advanced glycation end products-binding COOH-terminal motif of amphoterin inhibits invasive migration and metastasis. Cancer Res. 62, 4805–4811. 12183440

[B89] Hwang I. Park Y. J. Kim Y. R. Kim Y. N. Ka S. Lee H. Y.. (2015). Alteration of gut microbiota by vancomycin and bacitracin improves insulin resistance via glucagon-like peptide 1 in diet-induced obesity. FASEB J. 29, 2397–2411. 10.1096/fj.14-265983 25713030

[B90] Illien-Jünger S. Lu Y. Qureshi S. A. Hecht A. C. Cai W. Vlassara H.. (2015). Chronic ingestion of advanced glycation end products induces degenerative spinal changes and hypertrophy in aging pre-diabetic mice. PLoS ONE 10:e0116625. 10.1371/journal.pone.0116625 25668621 PMC4323205

[B91] Jeschke M. G. Klein D. Bolder U. Einspanier R. (2004). Insulin attenuates the systemic inflammatory response in endotoxemic rats. Endocrinology 145, 4084–4093. 10.1210/en.2004-0592 15192048

[B92] Jin Z. Mendu S. K. Birnir B. (2013). GABA is an effective immunomodulatory molecule. Amino Acids 45, 87–94. 10.1007/s00726-011-1193-7 22160261 PMC3680704

[B93] Jing G. Zhang M. (2011). Tang niao bing nao bing de yang hua ying ji sun shang ji zhi liao. Chin. J. Diabetes 19, 68–70.

[B94] Kamada H. Yu F. Nito C. Chan P. H. (2007). Influence of hyperglycemia on oxidative stress and matrix metalloproteinase-9 activation after focal cerebral ischemia/reperfusion in rats: relation to blood-brain barrier dysfunction. Stroke 38, 1044–1049. 10.1161/01.STR.0000258041.75739.cb 17272778 PMC1828129

[B95] Kamal A. Biessels G. J. Urban I. J. Gispen W. H. (1999). Hippocampal synaptic plasticity in streptozotocin-diabetic rats: impairment of long-term potentiation and facilitation of long-term depression. Neuroscience 90, 737–745. 10.1016/S0306-4522(98)00485-0 10218775

[B96] Kamboj S. S. Chopra K. Sandhir R. (2008). Neuroprotective effect of N-acetylcysteine in the development of diabetic encephalopathy in streptozotocin-induced diabetes. Metab. Brain Dis. 23, 427–443. 10.1007/s11011-008-9104-7 18802743

[B97] Kanoski S. E. Davidson T. L. (2010). Different patterns of memory impairments accompany short- and longer-term maintenance on a high-energy diet. J. Exp. Psychol. Anim. Behav. Process. 36, 313–319. 10.1037/a0017228 20384410

[B98] Kanoski S. E. Davidson T. L. (2011). Western diet consumption and cognitive impairment: links to hippocampal dysfunction and obesity. Physiol. Behav. 103, 59–68. 10.1016/j.physbeh.2010.12.003 21167850 PMC3056912

[B99] Kanoski S. E. Zhang Y. Zheng W. Davidson T. L. (2010). The effects of a high-energy diet on hippocampal function and blood-brain barrier integrity in the rat. J. Alzheimers Dis. 21, 207–219. 10.3233/JAD-2010-091414 20413889 PMC4975946

[B100] Karlsson F. H. Tremaroli V. Nookaew I. Bergström G. Behre C. J. Fagerberg B.. (2013). Gut metagenome in European women with normal, impaired and diabetic glucose control. Nature 498, 99–103. 10.1038/nature12198 23719380

[B101] Keller P. J. Le Van Q. Kim S. U. Bown D. H. Chen H. C. Kohnle A.. (1988). Biosynthesis of riboflavin: mechanism of formation of the ribitylamino linkage. Biochemistry 27, 1117–1120. 10.1021/bi00404a006 3130093

[B102] Kelly C. J. Zheng L. Campbell E. L. Saeedi B. Scholz C. C. Bayless A. J.. (2015). Crosstalk between microbiota-derived short-chain fatty acids and intestinal epithelial HIF augments tissue barrier function. Cell Host Microbe 17, 662–671. 10.1016/j.chom.2015.03.005 25865369 PMC4433427

[B103] Kiank C. Taché Y. Larauche M. (2010). Stress-related modulation of inflammation in experimental models of bowel disease and post-infectious irritable bowel syndrome: role of corticotropin-releasing factor receptors. Brain Behav. Immun. 24, 41–48. 10.1016/j.bbi.2009.08.006 19698778 PMC2962412

[B104] Kierdorf K. Wang Y. Neumann H. (2010). Immune-mediated CNS damage. Results Probl. Cell Differ. 51, 173–196. 10.1007/400_2008_15 19130024

[B105] Kim J. Jang H. S. Park K. M. (2010). Reactive oxygen species generated by renal ischemia and reperfusion trigger protection against subsequent renal ischemia and reperfusion injury in mice. Am. J. Physiol. Renal Physiol. 298, F158–F166. 10.1152/ajprenal.00474.2009 19864300

[B106] Kimura I. Inoue D. Maeda T. Hara T. Ichimura A. Miyauchi S.. (2011). Short-chain fatty acids and ketones directly regulate sympathetic nervous system via G protein-coupled receptor 41 (GPR41). Proc. Natl. Acad. Sci. U.S.A. 108, 8030–8035. 10.1073/pnas.1016088108 21518883 PMC3093469

[B107] Krabbe K. S. Reichenberg A. Yirmiya R. Smed A. Pedersen B. K. Bruunsgaard H. (2005). Low-dose endotoxemia and human neuropsychological functions. Brain Behav. Immun. 19, 453–460. 10.1016/j.bbi.2005.04.010 15963684

[B108] Kuhad A. Chopra K. (2007). Curcumin attenuates diabetic encephalopathy in rats: behavioral and biochemical evidences. Eur. J. Pharmacol. 576, 34–42. 10.1016/j.ejphar.2007.08.001 17822693

[B109] Lane-Donovan C. Philips G. T. Herz J. (2014). More than cholesterol transporters: lipoprotein receptors in CNS function and neurodegeneration. Neuron 83, 771–787. 10.1016/j.neuron.2014.08.005 25144875 PMC4240629

[B110] Layé S. Bluthé R. M. Kent S. Combe C. Médina C. Parnet P.. (1995). Subdiaphragmatic vagotomy blocks induction of IL-1 beta mRNA in mice brain in response to peripheral LPS. Am. J. Physiol. 268, R1327–R1331. 7771597 10.1152/ajpregu.1995.268.5.R1327

[B111] Leclercq S. Matamoros S. Cani P. D. Neyrinck A. M. Jamar F. Stärkel P.. (2014). Intestinal permeability, gut-bacterial dysbiosis, and behavioral markers of alcohol-dependence severity. Proc. Natl. Acad. Sci. U.S.A. 111, E4485–E4493. 10.1073/pnas.1415174111 25288760 PMC4210345

[B112] Leclercq S. de Timary P. Delzenne N. M. Stärkel P. (2017). The link between inflammation, bugs, the intestine and the brain in alcohol dependence. Transl. Psychiatry 7:e1048. 10.1038/tp.2017.15 28244981 PMC5545644

[B113] Levenson J. M. O'Riordan K. J. Brown K. D. Trinh M. A. Molfese D. L. Sweatt J. D. (2004). Regulation of histone acetylation during memory formation in the hippocampus. J. Biol. Chem. 279, 40545–40559. 10.1074/jbc.M402229200 15273246

[B114] Li L. Zhang Z. F. Holscher C. Gao C. Jiang Y. H. Liu Y. Z. (2012). (Val^8^) glucagon-like peptide-1 prevents tau hyperphosphorylation, impairment of spatial learning and ultra-structural cellular damage induced by streptozotocin in rat brains. Eur. J. Pharmacol. 674, 280–286. 10.1016/j.ejphar.2011.11.005 22115895

[B115] Li P. C. Liu L. F. Jou M. J. Wang H. K. (2016). The GLP-1 receptor agonists exendin-4 and liraglutide alleviate oxidative stress and cognitive and micturition deficits induced by middle cerebral artery occlusion in diabetic mice. BMC Neurosci. 17, 37. 10.1186/s12868-016-0272-9 27296974 PMC4907076

[B116] Li W. Dowd S. E. Scurlock B. Acosta-Martinez V. Lyte M. (2009). Memory and learning behavior in mice is temporally associated with diet-induced alterations in gut bacteria. Physiol. Behav. 96, 557–567. 10.1016/j.physbeh.2008.12.004 19135464

[B117] Li X. Hu J. Zhang Q. Sun X. Li S. (2009). Urocortin 1 improves renal function in rats with streptozotocin-induced diabetes by inhibiting overproduction of TGF-beta 1 and VEGF. Br. J. Pharmacol. 157, 994–1003. 10.1111/j.1476-5381.2009.00264.x 19466989 PMC2737658

[B118] Li X. Hu J. Zhang R. Sun X. Zhang Q. Guan X.. (2008). Urocortin ameliorates diabetic nephropathy in obese db/db mice. Br. J. Pharmacol. 154, 1025–1034. 10.1038/bjp.2008.155 18587447 PMC2451047

[B119] Liao F. Hori Y. Hudry E. Bauer A. Q. Jiang H. Mahan T. E.. (2014). Anti-ApoE antibody given after plaque onset decreases Aβ accumulation and improves brain function in a mouse model of Aβ amyloidosis. J. Neurosci. 34, 7281–7292. 10.1523/JNEUROSCI.0646-14.2014 24849360 PMC4028501

[B120] Liu D. Chan S. L. de Souza-Pinto N. C. Slevin J. R. Wersto R. P. Zhan M.. (2006). Mitochondrial UCP4 mediates an adaptive shift in energy metabolism and increases the resistance of neurons to metabolic and oxidative stress. Neuromolecular Med. 8, 389–414. 10.1385/NMM:8:3:389 16775390

[B121] Liu G. Chen L. Ran X. Han J. Zheng D. Chen Q. (2003). Expression of nitric oxide synthase in hippocampus of STZ-induced diabetic rats and relation between nitric oxide and cognitive function. J. Shandong Univ. 41, 171–174.

[B122] Liu X. Liu C. Zhang X. Zhao J. Xu J. (2015). Urocortin ameliorates diabetic cardiomyopathy in rats via the Akt/GSK-3β signaling pathway. Exp. Ther. Med. 9, 667–674. 10.3892/etm.2015.2211 25667611 PMC4316969

[B123] Liu X. Xiao Q. Zhao K. Gao Y. (2013). Ghrelin inhibits high glucose-induced PC12 cell apoptosis by regulating TLR4/NF-κB pathway. Inflammation 36, 1286–1294. 10.1007/s10753-013-9667-2 23813326

[B124] Loef M. Walach H. (2013). Midlife obesity and dementia: meta-analysis and adjusted forecast of dementia prevalence in the United States and China. Obesity 21, E51–E55. 10.1002/oby.20037 23401370

[B125] Lyte M. (2011). Probiotics function mechanistically as delivery vehicles for neuroactive compounds: microbial endocrinology in the design and use of probiotics. Bioessays 33, 574–581. 10.1002/bies.201100024 21732396

[B126] Macfabe D. F. (2012). Short-chain fatty acid fermentation products of the gut microbiome: implications in autism spectrum disorders. Microb. Ecol. Health Dis. 24:23 10.3402/mehd.v23i0.19260 PMC3747729 23990817

[B127] MacFabe D. F. Cain N. E. Boon F. Ossenkopp K. P. Cain D. P. (2011). Effects of the enteric bacterial metabolic product propionic acid on object-directed behavior, social behavior, cognition, and neuroinflammation in adolescent rats: relevance to autism spectrum disorder. Behav. Brain Res. 217, 47–54. 10.1016/j.bbr.2010.10.005 20937326

[B128] Macfarlane S. Macfarlane G. T. (2003). Regulation of short-chain fatty acid production. Proc. Nutr. Soc. 62, 67–72. 10.1079/PNS2002207 12740060

[B129] Manschot S. M. Biessels G. J. Cameron N. E. Cotter M. A. Kamal A. Kappelle L. J.. (2003). Angiotensin converting enzyme inhibition partially prevents deficits in water maze performance, hippocampal synaptic plasticity and cerebral blood flow in streptozotocin-diabetic rats. Brain Res. 966, 274–282. 10.1016/S0006-8993(02)04211-7 12618350

[B130] Márquez E. Riera M. Pascual J. Soler M. J. (2015). Renin-angiotensin system within the diabetic podocyte. Am. J. Physiol. Renal Physiol. 308, F1–F10. 10.1152/ajprenal.00531.2013 25339703

[B131] Maslowski K. M. Vieira A. T. Ng A. Kranich J. Sierro F. Yu D.. (2009). Regulation of inflammatory responses by gut microbiota and chemoattractant receptor GPR43. Nature 461, 1282–1286. 10.1038/nature08530 19865172 PMC3256734

[B132] Mastrocola R. Restivo F. Vercellinatto I. Danni O. Brignardello E. Aragno M.. (2005). Oxidative and nitrosative stress in brain mitochondria of diabetic rats. J. Endocrinol. 187, 37–44. 10.1677/joe.1.06269 16214939

[B133] Mattson M. P. Camandola S. (2001). NF-κB in neuronal plasticity and neurodegenerative disorders. J. Clin. Invest. 107, 247–254. 10.1172/JCI11916 11160145 PMC199201

[B134] Maulik M. Westaway D. Jhamandas J. H. Kar S. (2013). Role of cholesterol in APP metabolism and its significance in Alzheimer's disease pathogenesis. Mol. Neurobiol. 47, 37–63. 10.1007/s12035-012-8337-y 22983915

[B135] Mayer E. A. Padua D. Tillisch K. (2014). Altered brain-gut axis in autism: comorbidity or causative mechanisms? Bioessays 36, 933–939. 10.1002/bies.201400075 25145752

[B136] Migdalis I. N. Xenos K. Chairopoulos K. Varvarigos N. Leontiades E. Karmaniolas K. (2000). Ca(2+)-Mg(2+)-ATPase activity and ionized calcium in Type 2 diabetic patients with neuropathy. Diabetes Res. Clin. Pract. 49, 113–118. 10.1016/S0168-8227(00)00150-9 10963822

[B137] Miles W. R. Root H. F. (1922). Psychologic tests applied to diabetic patients. Arch. Intern. Med. 30, 767–777. 10.1001/archinte.1922.00110120086003

[B138] Morgan M. Y. (1991). The treatment of chronic hepatic encephalopathy. Hepatogastroenterology 38, 377–387. 1662661

[B139] Muranyi M. Fujioka M. He Q. Han A. Yong G. Csiszar K.. (2003). Diabetes activates cell death pathway after transient focal cerebral ischemia. Diabetes 52, 481–486. 10.2337/diabetes.52.2.481 12540624

[B140] Nguyen J. C. Killcross A. S. Jenkins T. A. (2014). Obesity and cognitive decline: role of inflammation and vascular changes. Front. Neurosci. 8:375. 10.3389/fnins.2014.00375 25477778 PMC4237034

[B141] Noble E. E. Hsu T. M. Jones R. B. Fodor A. A. Goran M. I. Kanoski S. E. (2017). Early-life sugar consumption affects the rat microbiome independently of obesity. J. Nutr. 147, 20–28. 10.3945/jn.116.238816 27903830 PMC5177734

[B142] Noell S. Fallier-Becker P. Deutsch U. Mack A. F. Wolburg H. (2009). Agrin defines polarized distribution of orthogonal arrays of particles in astrocytes. Cell Tissue Res. 337, 185–195. 10.1007/s00441-009-0812-z 19449033

[B143] Obrenovich M. Flückiger R. Sykes L. Donskey C. (2016). The co-metabolism within the gut-brain metabolic interaction: potential targets for drug treatment and design. CNS Neurol. Disord. Drug Targets 15, 127–134. 10.2174/1871527315666160202123107 26831263

[B144] Ojo B. El-Rassi G. D. Payton M. E. Perkins-Veazie P. Clarke S. Smith B. J.. (2016). Mango supplementation modulates gut microbial dysbiosis and short-chain fatty acid production independent of body weight reduction in C57BL/6 mice fed a high-fat diet. J. Nutr. 146, 1483–1491. 10.3945/jn.115.226688 27358411

[B145] Poduslo J. F. Curran G. L. Berg C. T. (1994). Macromolecular permeability across the blood-nerve and blood-brain barriers. Proc. Natl. Acad. Sci. U.S.A. 91, 5705–5709. 10.1073/pnas.91.12.5705 8202551 PMC44065

[B146] Posse de Chaves E. (2012). Reciprocal regulation of cholesterol and beta amyloid at the subcellular level in Alzheimer's disease. Can. J. Physiol. Pharmacol. 90, 753–764. 10.1139/y2012-076 22626060

[B147] Qin J. Li Y. Cai Z. Li S. Zhu J. Zhang F.. (2012). A metagenome-wide association study of gut microbiota in type 2 diabetes. Nature 490, 55–60. 10.1038/nature11450 23023125

[B148] Quigley E. M. (2008). Probiotics in functional gastrointestinal disorders: what are the facts? Curr. Opin. Pharmacol. 8, 704–708. 10.1016/j.coph.2008.08.007 18775516

[B149] Rakoff-Nahoum S. Paglino J. Eslami-Varzaneh F. Edberg S. Medzhitov R. (2004). Recognition of commensal microflora by toll-like receptors is required for intestinal homeostasis. Cell 118, 229–241. 10.1016/j.cell.2004.07.002 15260992

[B150] Reolon G. K. Maurmann N. Werenicz A. Garcia V. A. Schröder N. Wood M. A.. (2011). Posttraining systemic administration of the histone deacetylase inhibitor sodium butyrate ameliorates aging-related memory decline in rats. Behav. Brain Res. 221, 329–332. 10.1016/j.bbr.2011.03.033 21421011 PMC3093300

[B151] Reske-Nielsen E. Lundbæk K. Rafaelsen O. J. (1966). Pathological changes in the central and peripheral nervous system of young long-term diabetics: I. Diabetic encephalopathy. Diabetologia 1, 233–241. 24173307 10.1007/BF01257917

[B152] Rhee S. H. Pothoulakis C. Mayer E. A. (2009). Principles and clinical implications of the brain-gut-enteric microbiota axis. Nat. Rev. Gastroenterol. Hepatol. 6, 306–314. 10.1038/nrgastro.2009.35 19404271 PMC3817714

[B153] Rönnemaa E. Zethelius B. Sundelöf J. Sundström J. Degerman-Gunnarsson M. Berne C.. (2008). Impaired insulin secretion increases the risk of Alzheimer disease. Neurology. 71, 1065–1071. 10.1212/01.wnl.0000310646.32212.3a 18401020

[B154] Roustit M. M. Vaughan J. M. Jamieson P. M. Cleasby M. E. (2014). Urocortin 3 activates AMPK and AKT pathways and enhances glucose disposal in rat skeletal muscle. J. Endocrinol. 223, 143–154. 10.1530/JOE-14-0181 25122003 PMC4191181

[B155] Samuel B. S. Shaito A. Motoike T. Rey F. E. Backhed F. Manchester J. K.. (2008). Effects of the gut microbiota on host adiposity are modulated by the short-chain fatty-acid binding G protein-coupled receptor, Gpr41. Proc. Natl. Acad. Sci. U.S.A. 105, 16767–16772. 10.1073/pnas.0808567105 18931303 PMC2569967

[B156] Saulnier D. M. Ringel Y. Heyman M. B. Foster J. A. Bercik P. Shulman R. J.. (2013). The intestinal microbiome, probiotics and prebiotics in neurogastroenterology. Gut Microbes 4, 17–27. 10.4161/gmic.22973 23202796 PMC3555881

[B157] Schedlowski M. Engler H. Grigoleit J. S. (2014). Endotoxin-induced experimental systemic inflammation in humans: a model to disentangle immune-to-brain communication. Brain Behav. Immun. 35, 1–8. 10.1016/j.bbi.2013.09.015 24491305

[B158] Schreibelt G. Kooij G. Reijerkerk A. van Doorn R. Gringhuis S. I. van der Pol S.. (2007). Reactive oxygen species alter brain endothelial tight junction dynamics via RhoA, PI3 kinase, and PKB signaling. FASEB J. 21, 3666–3676. 10.1096/fj.07-8329com 17586731

[B159] Segain J. P. Raingeard de La Blétière D. Bourreille A. Leray V. Gervois N.. (2000). Butyrate inhibits inflammatory responses through NFκB inhibition: implications for Crohn's disease. Gut 47, 397–403. 10.1136/gut.47.3.397 10940278 PMC1728045

[B160] Sellbom K. S. Gunstad J. (2012). Cognitive function and decline in obesity. J. Alzheimers Dis. 30, S89–S95. 10.3233/JAD-2011-111073 22258511

[B161] Shimizu F. Sano Y. Haruki H. Kanda T. (2011). Advanced glycation end-products induce basement membrane hypertrophy in endoneurial microvessels and disrupt the blood-nerve barrier by stimulating the release of TGF-β and vascular endothelial growth factor (VEGF) by pericytes. Diabetologia 54, 1517–1526. 10.1007/s00125-011-2107-7 21409414

[B162] Shimizu F. Sano Y. Tominaga O. Maeda T. Abe M. A. Kanda T. (2013). Advanced glycation end-products disrupt the blood-brain barrier by stimulating the release of transforming growth factor-β by pericytes and vascular endothelial growth factor and matrix metalloproteinase-2 by endothelial cells *in vitro*. Neurobiol. Aging 34, 1902–1912. 10.1016/j.neurobiolaging.2013.01.012 23428182

[B163] Singh R. Barden A. Mori T. Beilin L. (2001). Advanced glycation end-products: a review. Diabetologia 44, 129–146. 10.1007/s001250051591 11270668

[B164] Singhal K. Sandhir R. (2015). L-type calcium channel blocker ameliorates diabetic encephalopathy by modulating dysregulated calcium homeostasis. J. Neurosci. Res. 93, 296–308. 10.1002/jnr.23478 25267297

[B165] Singh-Manoux A. Dugravot A. Brunner E. Kumari M. Shipley M. Elbaz A.. (2014). Interleukin-6 and C-reactive protein as predictors of cognitive decline in late midlife. Neurology 83, 486–493. 10.1212/WNL.0000000000000665 24991031 PMC4141998

[B166] Smith M. A. Taneda S. Richey P. L. Miyata S. Yan S. D. Stern D.. (1994). Advanced Maillard reaction end products are associated with Alzheimer disease pathology. Proc. Natl. Acad. Sci. U.S.A. 91, 5710–5714. 10.1073/pnas.91.12.5710 8202552 PMC44066

[B167] Söderholm J. D. Yates D. A. Gareau M. G. Yang P. C. MacQueen G. Perdue M. H. (2002). Neonatal maternal separation predisposes adult rats to colonic barrier dysfunction in response to mild stress. Am. J. Physiol. Gastrointest. Liver Physiol. 283, G1257–G1263. 10.1152/ajpgi.00314.2002 12388189

[B168] Steenbergen L. Sellaro R. van. Hemert S. Bosch J. A. Colzato L. S. (2015). A randomized controlled trial to test the effect of multispecies probiotics on cognitive reactivity to sad mood. Brain Behav. Immun. 48, 258–264. 10.1016/j.bbi.2015.04.003 25862297

[B169] Stefanko D. P. Barrett R. M. Ly A. R. Reolon G. K. Wood M. A. (2009). Modulation of long-term memory for object recognition via HDAC inhibition. Proc. Natl. Acad. Sci. U.S.A. 106, 9447–9452. 10.1073/pnas.0903964106 19470462 PMC2695069

[B170] Steiner E. Enzmann G. U. Lyck R. Lin S. Rüegg M. A. Kröger S.. (2014). The heparan sulfate proteoglycan agrin contributes to barrier properties of mouse brain endothelial cells by stabilizing adherens junctions. Cell Tissue Res. 358, 465–479. 10.1007/s00441-014-1969-7 25107608 PMC4210653

[B171] Stilling R. M. Dinan T. G. Cryan J. F. (2014). Microbial genes, brain & behaviour - epigenetic regulation of the gut-brain axis. Genes Brain Behav. 13, 69–86. 10.1111/gbb.12109 24286462

[B172] Stranahan A. M. Hao S. Dey A. Yu X. Baban B. (2016). Blood-brain barrier breakdown promotes macrophage infiltration and cognitive impairment in leptin receptor-deficient mice. J. Cereb. Blood Flow Metab. 36, 2108–2121. 10.1177/0271678x16642233 27034250 PMC5363667

[B173] Suárez-Zamorano N. Fabbiano S. Chevalier C. Stojanović O. Colin D. J. Stevanović A.. (2015). Microbiota depletion promotes browning of white adipose tissue and reduces obesity. Nat. Med. 21, 1497–1501. 10.1038/nm.3994 26569380 PMC4675088

[B174] Sudo N. Chida Y. Aiba Y. Sonoda J. Oyama N. Yu X. N.. (2004). Postnatal microbial colonization programs the hypothalamic-pituitary-adrenal system for stress response in mice. J. Physiol. 558, 263–275. 10.1113/jphysiol.2004.063388 15133062 PMC1664925

[B175] Sugimoto K. Nishizawa Y. Horiuchi S. Yagihashi S. (1997). Localization in human diabetic peripheral nerve of N(ε)-carboxymethyllysine-protein adducts, an advanced glycation endproduct. Diabetologia 40, 1380–1387. 10.1007/s001250050839 9447944

[B176] Sun J. Wang F. Li H. Zhang H. Jin J. Chen W.. (2015). Neuroprotective effect of sodium butyrate against cerebral ischemia/reperfusion injury in mice. Biomed. Res. Int. 2015:395895. 10.1155/2015/395895 26064905 PMC4439479

[B177] Suzuki T. Yoshida S. Hara H. (2008). Physiological concentrations of short-chain fatty acids immediately suppress colonic epithelial permeability. Br. J. Nutr. 100, 297–305. 10.1017/S0007114508888733 18346306

[B178] Sveen K. A. Karimé B. Jørum E. Mellgren S. I. Fagerland M. W. Monnier V. M.. (2013). Small- and large-fiber neuropathy after 40 years of type 1 diabetes: associations with glycemic control and advanced protein glycation: the Oslo Study. Diab. Care 36, 3712–3717. 10.2337/dc13-0788 24026557 PMC3816884

[B179] Takeda A. Yasuda T. Miyata T. Goto Y. Wakai M. Watanabe M.. (1998). Advanced glycation end products co-localized with astrocytes and microglial cells in Alzheimer's disease brain. Acta Neuropathol. 95, 555–558. 10.1007/s004010050839 9650745

[B180] Talarowska M. Gałecki P. Maes M. Orzechowska A. Chamielec M. Bartosz G.. (2012). Nitric oxide plasma concentration associated with cognitive impairment in patients with recurrent depressive disorder. Neurosci. Lett. 510, 127–131. 10.1016/j.neulet.2012.01.018 22273980

[B181] Tao S. Liang X. Ying-Ge H. Yan W. Yi-Xiang S. Wei-Wei L. (2013). The abnormally high level of uric D-ribose for type-2 diabetics. Prog. Biochem. Biophys. 40, 816–825. 10.3724/SP.J.1206.2013.00341

[B182] Tilling T. Engelbertz C. Decker S. Korte D. Hüwel S. Galla H. J. (2002). Expression and adhesive properties of basement membrane proteins in cerebral capillary endothelial cell cultures. Cell Tissue Res. 310, 19–29. 10.1007/s00441-002-0604-1 12242480

[B183] Tirabassi G. Corona G. Lamonica G. R. Lenzi A. Maggi M. Balercia G. (2016). Diabetes mellitus-associated functional hypercortisolism impairs sexual function in male late-onset hypogonadism. Horm. Metab. Res. 48, 48–53. 10.1055/s-0035-1548870 25951320

[B184] Tokuno S. Chen F. Pernow J. Jiang J. Valen G. (2002). Effects of spontaneous or induced brain ischemia on vessel reactivity: the role of inducible nitric oxide synthase. Life Sci. 71, 679–692. 10.1016/S0024-3205(02)01711-3 12072156

[B185] Tolhurst G. Heffron H. Lam Y. S. Parker H. E. Habib A. M. Diakogiannaki E.. (2012). Short-chain fatty acids stimulate glucagon-like peptide-1 secretion via the G-protein-coupled receptor FFAR2. Diabetes 61, 364–371. 10.2337/db11-1019 22190648 PMC3266401

[B186] Toth C. Schmidt A. M. Tuor U. I. Francis G. Foniok T. Brussee V.. (2006). Diabetes, leukoencephalopathy and rage. Neurobiol. Dis. 23, 445–461. 10.1016/j.nbd.2006.03.015 16815028

[B187] Trompette A. Gollwitzer E. S. Yadava K. Sichelstiel A. K. Sprenger N. Ngom-Bru C.. (2014). Gut microbiota metabolism of dietary fiber influences allergic airway disease and hematopoiesis. Nat. Med. 20, 159–166. 10.1038/nm.3444 24390308

[B188] Uribarri J. Cai W. Ramdas M. Goodman S. Pyzik R. Chen X.. (2011). Restriction of advanced glycation end products improves insulin resistance in human type 2 diabetes: potential role of AGER1 and SIRT1. Diab. Care 34, 1610–1616. 10.2337/dc11-0091 21709297 PMC3120204

[B189] van den Berg E. Reijmer Y. D. de Bresser J. Kessels R. P. Kappelle L. J. Biessels G. J.. (2010). A 4 year follow-up study of cognitive functioning in patients with type 2 diabetes mellitus. Diabetologia 53, 58–65. 10.1007/s00125-009-1571-9 19882137 PMC2789935

[B190] van der Meulen T. Donaldson C. J. Cáceres E. Hunter A. E. Cowing-Zitron C. Pound L. D.. (2015). Urocortin3 mediates somatostatin-dependent negative feedback control of insulin secretion. Nat. Med. 21, 769–776. 10.1038/nm.3872 26076035 PMC4496282

[B191] Venereau E. Schiraldi M. Uguccioni M. Bianchi M. E. (2013). HMGB1 and leukocyte migration during trauma and sterile inflammation. Mol. Immunol. 55, 76–82. 10.1016/j.molimm.2012.10.037 23207101

[B192] Vlassara H. Palace M. R. (2003). Glycoxidation: the menace of diabetes and aging. Mt. Sinai J. Med. 70, 232–241. 12968196

[B193] Vlassara H. Striker G. E. (2011). AGE restriction in diabetes mellitus: a paradigm shift. Nat. Rev. Endocrinol. 7, 526–539. 10.1038/nrendo.2011.74 21610689 PMC3708644

[B194] Vrieze A. Van Nood E. Holleman F. Salojärvi J. Kootte R. S. Bartelsman J. F.. (2012). Transfer of intestinal microbiota from lean donors increases insulin sensitivity in individuals with metabolic syndrome. Gastroenterology. 143, 913–916.e7. 10.1053/j.gastro.2012.06.031 22728514

[B195] Wang B. Miao Y. Zhao Z. Zhong Y. (2015). Inflammatory macrophages promotes development of diabetic encephalopathy. Cell Physiol. Biochem. 36, 1142–1150. 10.1159/000430285 26113412

[B196] Wang D. S. Zurek A. A. Lecker I. Yu J. Abramian A. M. Avramescu S.. (2012). Memory deficits induced by inflammation are regulated by α5-subunit-containing GABAA receptors. Cell Rep. 2, 488–496. 10.1016/j.celrep.2012.08.022 22999935 PMC4391624

[B197] Wautier J. L. Guillausseau P. J. (1998). Diabetes, advanced glycation endproducts and vascular disease. Vasc. Med. 3, 131–137. 10.1177/1358836X9800300207 9796076

[B198] Wei Y. Han C. S. Zhou J. Liu Y. Chen L. He R. Q. (2012). D-ribose in glycation and protein aggregation. Biochim. Biophys. Acta 1820, 488–494. 10.1016/j.bbagen.2012.01.005 22274132

[B199] Wichmann M. A. Cruickshanks K. J. Carlsson C. M. Chappell R. Fischer M. E. Klein B. E.. (2014). Long-term systemic inflammation and cognitive impairment in a population-based cohort. J. Am. Geriatr. Soc. 62, 1683–1691. 10.1111/jgs.12994 25123210 PMC4196672

[B200] Windham B. G. Simpson B. N. Lirette S. Bridges J. Bielak L. Peyser P. A.. (2014). Associations between inflammation and cognitive function in African Americans and European Americans. J. Am. Geriatr. Soc. 62, 2303–2310. 10.1111/jgs.13165 25516026 PMC4270090

[B201] Wisse L. E. de Bresser J. Geerlings M. I. Reijmer Y. D. Portegies M. L. Brundel M.. (2014). Global brain atrophy but not hippocampal atrophy is related to type 2 diabetes. J. Neurol. Sci. 344, 32–36. 10.1016/j.jns.2014.06.008 24958596

[B202] Wood W. G. Li L. Müller W. E. Eckert G. P. (2014). Cholesterol as a causative factor in Alzheimer's disease: a debatable hypothesis. J. Neurochem. 129, 559–572. 10.1111/jnc.12637 24329875 PMC3999290

[B203] Wu X. Chen P. S. Dallas S. Wilson B. Block M. L. Wang C. C.. (2008). Histone deacetylase inhibitors up-regulate astrocyte GDNF and BDNF gene transcription and protect dopaminergic neurons. Int. J. Neuropsychopharmacol. 11, 1123–1134. 10.1017/S1461145708009024 18611290 PMC2579941

[B204] Wu X. Ma C. Han L. Nawaz M. Gao F. Zhang X.. (2010). Molecular characterisation of the faecal microbiota in patients with type II diabetes. Curr. Microbiol. 61, 69–78. 10.1007/s00284-010-9582-9 20087741

[B205] Wu Y. Xu Y. Zhou H. Tao J. Li S. (2006). Expression of urocortin in rat lung and its effect on pulmonary vascular permeability. J. Endocrinol. 189, 167–178. 10.1677/joe.1.06607 16614391

[B206] Xu Y. Wang S. Feng L. Zhu Q. Xiang P. He B. (2010). Blockade of PKC-beta protects HUVEC from advanced glycation end products induced inflammation. Int. Immunopharmacol. 10, 1552–1559. 10.1016/j.intimp.2010.09.006 20875828

[B207] Xue H. Y. Jin L. Jin L. J. Li X. Y. Zhang P. Ma Y. S.. (2009). Aucubin prevents loss of hippocampal neurons and regulates antioxidative activity in diabetic encephalopathy rats. Phytother. Res. 23, 980–986. 10.1002/ptr.2734 19140154

[B208] Yang S. N. Tang Y. G. Zucker R. S. (1999). Selective induction of LTP and LTD by postsynaptic [Ca2+]i elevation. J. Neurophysiol. 81, 781–787. 10036277 10.1152/jn.1999.81.2.781

[B209] Yoo D. Y. Kim D. W. Kim M. J. Choi J. H. Jung H. Y. Nam S. M.. (2015). Sodium butyrate, a histone deacetylase Inhibitor, ameliorates SIRT2-induced memory impairment, reduction of cell proliferation, and neuroblast differentiation in the dentate gyrus. Neurol. Res. 37, 69–76. 10.1179/1743132814Y.0000000416 24963697

[B210] Yoo D. Y. Kim W. Nam S. M. Kim D. W. Chung J. Y. Choi S. Y.. (2011). Synergistic effects of sodium butyrate, a histone deacetylase inhibitor, on increase of neurogenesis induced by pyridoxine and increase of neural proliferation in the mouse dentate gyrus. Neurochem. Res. 36, 1850–1857. 10.1007/s11064-011-0503-5 21597935

[B211] Yoo D. Y. Yim H. S. Jung H. Y. Nam S. M. Kim J. W. Choi J. H.. (2016). Chronic type 2 diabetes reduces the integrity of the blood-brain barrier by reducing tight junction proteins in the hippocampus. J. Vet. Med. Sci. 78, 957–962. 10.1292/jvms.15-0589 26876499 PMC4937155

[B212] Yu X. Xu X. Jackson A. Sun J. Huang P. Mao Y.. (2016). Blood brain barrier disruption in diabetic stroke related to unfavorable outcome. Cerebrovasc. Dis. 42, 49–56. 10.1159/000444809 26986824

[B213] Yudkin J. S. Stehouwer C. D. Emeis J. J. Coppack S. W. (1999). C-reactive protein in healthy subjects: associations with obesity, insulin resistance, and endothelial dysfunction: a potential role for cytokines originating from adipose tissue? Arterioscler. Thromb. Vasc. Biol. 19, 972–978. 10.1161/01.ATV.19.4.972 10195925

[B214] Zhao Q. Xiong Y. Ding D. Guo Q. Hong Z. (2012). Synergistic effect between apolipoprotein E ε4 and diabetes mellitus for dementia: result from a population-based study in urban China. J. Alzheimers Dis. 32, 1019–1027. 10.3233/JAD-2012-120442 22890095

[B215] Zhao W. Q. Alkon D. L. (2001). Role of insulin and insulin receptor in learning and memory. Mol. Cell Endocrinol. 177, 125–134. 10.1016/S0303-7207(01)00455-5 11377828

[B216] Zhu H. Huang Q. Xu H. Niu L. Zhou J. N. (2009). Antidepressant-like effects of sodium butyrate in combination with estrogen in rat forced swimming test: involvement of 5-HT(1A) receptors. Behav. Brain Res. 196, 200–206. 10.1016/j.bbr.2008.08.039 18817816

